# QTL Analyses in Multiple Populations Employed for the Fine Mapping and Identification of Candidate Genes at a Locus Affecting Sugar Accumulation in Melon (*Cucumis melo* L.)

**DOI:** 10.3389/fpls.2017.01679

**Published:** 2017-09-26

**Authors:** Jason M. Argyris, Aurora Díaz, Valentino Ruggieri, Marta Fernández, Torben Jahrmann, Yves Gibon, Belén Picó, Ana M. Martín-Hernández, Antonio J. Monforte, Jordi Garcia-Mas

**Affiliations:** ^1^Centre for Research in Agricultural Genomics (CSIC-IRTA-UAB-UB), Barcelona, Spain; ^2^Institut de Recerca i Tecnologia Agroalimentàries, Barcelona, Spain; ^3^Instituto de Biología Molecular y Celular de Plantas, Universitat Politècnica de València-Consejo Superior de Investigaciones Científicas, Valencia, Spain; ^4^Semillas Fitó S.A., Cabrera de Mar, Spain; ^5^UMR1332 Biologie du Fruit et Pathologie, Plateforme Métabolome Bordeaux, INRA, University of Bordeaux, Villenave d'Ornon, France; ^6^Institute for the Conservation and Breeding of the Agricultural Biodiversity, Universitat Politècnica de València (COMAV-UPV), Valencia, Spain

**Keywords:** QTL, melon, sugar, sucrose, NILs, fine-mapping, candidate genes, BEL1-like

## Abstract

Sugar content is the major determinant of both fruit quality and consumer acceptance in melon (*Cucumis melo* L), and is a primary target for crop improvement. Near-isogenic lines (NILs) derived from the intraspecific cross between a “Piel de Sapo” (PS) type and the exotic cultivar “Songwhan Charmi” (SC), and several populations generated from the cross of PS × Ames 24294 (“Trigonus”), a wild melon, were used to identify QTL related to sugar and organic acid composition. Seventy-eight QTL were detected across several locations and different years, with three important clusters related to sugar content located on chromosomes 4, 5, and 7. Two PS × SC NILs (SC5-1 and SC5-2) sharing a common genomic interval of 1.7 Mb at the top of chromosome 5 contained QTL reducing soluble solids content (SSC) and sucrose content by an average of 29 and 68%, respectively. This cluster collocated with QTL affecting sugar content identified in other studies in lines developed from the PS × SC cross and supported the presence of a stable consensus locus involved in sugar accumulation that we named *SUCQSC5.1*. QTL reducing soluble solids and sucrose content identified in the “Trigonus” mapping populations, as well as QTL identified in previous studies from other ssp. *agrestis* sources, collocated with *SUCQSC5.1*, suggesting that they may be allelic and implying a role in domestication. In subNILs derived from the PS × SC5-1 cross, *SUCQSC5.1* reduced SSC and sucrose content by an average of 18 and 34%, respectively, and was fine-mapped to a 56.1 kb interval containing four genes. Expression analysis of the candidate genes in mature fruit showed differences between the subNILs with PS alleles that were “high” sugar and SC alleles of “low” sugar phenotypes for MELO3C014519, encoding a putative BEL1-like homeodomain protein. Sequence differences in the gene predicted to affect protein function were restricted to SC and other ssp. *agrestis* cultivar groups. These results provide the basis for further investigation of genes affecting sugar accumulation in melon.

## Introduction

Melon (*Cucumis melo* L.) is a highly diversified and economically important crop species that is cultivated in temperate regions throughout the world. As melon fruits are mainly consumed for their sweet taste, sugar content is the major determinant of both consumer acceptance and fruit quality, and is a primary target for crop improvement. The major sugar that accumulates during fruit ripening is sucrose (Burger et al., 2003). Like other cucurbits, melon is a symplastic phloem loading species that exports the raffinose family oligosaccharides (RFOs) raffinose and stachyose, as well as sucrose, from source leaves to sink tissues such as developing fruits (Zhang et al., 2012). Sucrose accumulation is developmentally controlled by metabolism of carbohydrates occurring in the fruit sink (Hubbard et al., 1989). The metabolic pathway of carbohydrate metabolism has been elucidated, and comprises at least a dozen enzymatic reactions starting with the translocated RFOs and ending with sucrose metabolism and accumulation (Gao et al., 1999). Sucrose accumulation begins as fruit undergoes a metabolic change in the transition from fruit growth (Burger and Schaffer, 2007). This is characterized at the transcriptional level by expression of distinct groups of genes from one stage to the other (Dai et al., 2011).

*C. melo* is divided into two subspecies that are generally distinct in their geographical distribution: ssp. *melo*, which is found from India to Europe and America and comprises Occidental cultivars like cantaloupe, galia, honeydew, Western shippers, “Piel de Sapo,” and Christmas melon; and ssp. *agrestis* found from India to Japan and comprising Oriental cultivars (Monforte et al., 2014). The subspecies are split into15 cultivated botanical groups plus wild melons present in Africa and Asia (Pitrat, 2008; Esteras et al., 2013). There are varying degrees of genetic admixture between groups (Leida et al., 2015). Considerable natural variation in sucrose content exists among the subspecies and groups, from the non-sweet melons (e.g., *flexuosus* group that are eaten as a vegetable) to very sweet (e.g., *inodorus* and *cantalupensis* groups) (Stepansky et al., 1999). Genetic and genomic resources developed in melon over the past several years (Argyris et al., 2015a) together with different mapping populations have facilitated the development of a consensus genetic map (Diaz et al., 2011), and the identification of a number of quantitative trait loci (QTL) related to sugar accumulation (Monforte et al., 2004; Paris et al., 2008; Park et al., 2009; Harel-Beja et al., 2010; Perpiña et al., 2016; Castro et al., 2017).

A valuable resource for QTL mapping are near-isogenic lines (NILs), which contain a single homozygous introgression of a donor line in the genetic background of a recipient line (Eshed and Zamir, 1995). NILs are a powerful tool that have advantages over other types of immortal mapping populations in making possible the detection and estimation of QTL of small effect (Keurentjes et al., 2007). In melon, a set of 57 melon NILs was developed from the highly polymorphic intraspecific cross between a “Piel de Sapo” (PS) type (*C. melo* ssp. *melo* group *inodorus*) “T111” and the exotic cultivar “Songwhan Charmi” (SC) (*C. melo* ssp. *agrestis* group *conomon* accession PI 161375) (Eduardo et al., 2005). Recently, melon introgression line (IL) collections containing chromosomal segments of the Japanese melon “Ginsen Makuwa” (*C. melo* ssp. *agrestis* group *makuwa*) in the French Charentais-type “Vedrantais” (*C. melo* ssp. *melo* group *cantalupensis*) genetic background, and another containing segments of *C. melo* ssp. *agrestis* group *dudaim* in the PS background, have been developed (Perpiña et al., 2016; Castro et al., 2017). Among other traits, QTL affecting sugar content were reported in both cases.

Dissection of QTL identified in NILs through development of subNILs has been utilized to effectively map and clone QTL involved in fruit morphology (Fernandez-Silva et al., 2010), and fruit ripening (Rios et al., 2017) in melon. To date, fine-mapping of QTL involved in sugar accumulation has not been reported. Despite this, there is correspondence of positions of QTL in different mapping populations, with clustering of QTL for SSC and soluble sugars identified on chromosomes 2, 3, and 5 (Diaz et al., 2011). This suggests that some genetic mechanisms governing sugar content are conserved across distinct germplasm. However, individual QTL or collinear clusters of QTL generally fail to collocate with candidate genes encoding enzymes involved in sugar metabolism (Harel-Beja et al., 2010; Diaz et al., 2015) which implies a role for other structural or regulatory genes in this process instead. For example, a GWAS study in tomato, which serves as a model species for sugar accumulation in fleshy fruit, identified up to 16 SNP/candidate loci associations for SSC or soluble sugars, many of which were for unknown or unexpected genes not associated with carbohydrate metabolism (Sauvage et al., 2014). In other instances, a vacuolar processing enzyme (VPE) participating in protein maturation in the vacuole, and an auxin response factor regulating chlorophyll content in fruit, have also been shown to affect sugar accumulation in tomato (Ariizumi et al., 2011; Sagar et al., 2013). These examples highlight the important contribution of other physiological processes and metabolic pathways affecting this trait.

The PS × SC NILs have been used to detect QTL for SSC, soluble sugars and sugar derivatives (Eduardo et al., 2007; Obando-Ulloa et al., 2009). In both cases, the phenotypic evaluations were performed with a smaller, non-redundant set of NILs; and in the latter case, in just a single environment. Given the low stability of sugar QTL in the population (Eduardo et al., 2007) a comprehensive analysis in multiple environments with the full set of NILs was warranted. In order to identify QTL with stable effects and gain more insight on the genetic factors involved in sugar accumulation in melon fruits, we studied sugar content in the full set of the PS × SC NILs in three environments, and in several different populations derived from the cross between the same PS cultivar and the wild melon accession “Trigonus” (TRI) accession Ames 24294 (*C. melo* ssp. *agrestis* group *agrestis*) that was developed recently (Diaz et al., 2017).

## Materials and methods

### Plant material and phenotyping

The set of 47 melon NILs derived from the PS × SC cross used in this study were developed as described previously (Eduardo et al., 2005). The NILs and parental lines were evaluated in three locations in the spring/summer cycle in 2011 and 2012. At the COMAV-UPV (VAL11), Spain (39°28′11″ N—0°22′38″ W), the NILs and parental lines were grown in a greenhouse in 2011. Five plants per NIL were grown in a completely randomized design to produce two fruits per plant. The NILs and parental lines were grown again in the summer of 2012 at the IRTA research station in Caldes de Montbui (CDM) (41°37′54″ N—2°10′0.73″ W) and at Semillas Fitó S.A. in Cabrera de Mar (CAM) (41°31′41″ N—2°23′34.8″ W), Barcelona, Spain. In both trials, five plants of each NIL were transplanted at the three-leaf stage to the greenhouse in a randomized complete block design consisting of eight blocks with the NILs and the parental lines randomized within each block. Flowers were hand pollinated and each plant was pruned and allowed to set a single fruit. In CAM, lines producing at least two fruits were included in statistical analyses.

Fruits were harvested at 55 days after pollination (DAP) in all locations and data for weight, length and width were collected. Soluble solids content (SSC) (°Brix) was measured by first cutting a 2 cm radial section from around the center of the melon fruit perpendicular to the longitudinal axis, and then taking four 1 cm diameter core samples equidistant from around the melon ring. The core samples were homogenized and the juice extracted and analyzed with a digital hand-held refractometer (Atago Co. Ltd., Tokyo, Japan). A second set of four core samples was taken and each core was then cut into three equal sized pieces. In order to have a sample of flesh from each of the sectors of the melon fruit, single pieces of each of the four cores were combined into one sample, then placed into plastic vials, flash frozen in liquid nitrogen, and stored at −80°C. Preparation of frozen samples for analyses of soluble sugars sucrose (SUC), glucose (GLU), and fructose (FRU) and the organic acids citrate (CIT), and malate (MAL) is detailed in Perpiña et al. (2016). Metabolite extraction and measurement for two technical replicates for each biological replicate fruit from VAL (*n* = 152) and one technical replicate for each biological replicate fruit from CDM (*n* = 94) were as described previously (Jelitto et al., 1992; Hendriks et al., 2003).

Construction of the PS × TRI mapping populations are described in detail elsewhere (Diaz et al., 2017). Briefly, a F_2_ population of 200 plants was acclimated and transplanted for three independent experiments in the same facilities described above at COMAV-UPV in 2011 (VAL11) and Zaragoza (Centro de Investigación y Tecnología Agroalimentaria de Aragón, CITA, 41° 43′26″ N—0° 48′ 31″ O) in 2011 and 2012 (Z11 and Z12). In VAL11, a single plant of each of a subset of 113 cloned F_2_ plants was grown in the greenhouse in drip-irrigated pots and hand pollinated, following a full randomized design, whereas in ZA11 and ZA12 the replicates consisted in one plot with three plants grown in open field also randomized and open-pollinated.

Backcross populations (toward PS) were obtained (BC1 and BC2) and grown in the greenhouse in COMAV-UPV in 2010 (VAL10) and 2012 (VAL12) as stated before. SSC was evaluated in all trials and sugar analysis in the VAL10 and VAL12 trials.

The line TRI5-1 was developed from a BC3 line by marker assisted selection and introgression of a segment of chromosome 5 from TRI into the PS genetic background followed by two generations of selfing to obtain a BC3S2 IL. Ten plants of TRI5-1 were grown in CDM14 and VAL14. SSC and soluble sugars content were evaluated in both trials.

### Phenotypic data analysis

Statistical analysis of SSC, sugar, and organic acid content was performed using JMP (version 8.0.1 for Windows, SAS institute, Cary, N.C.) and SAS (SAS institute, Cary, N.C.) for both the SC NILs and TRI-derived lines. For the SC NILs, a one-way analysis of variance of data was conducted using SAS PROC ANOVA with the *hovtest* option of the MEANS statement to test for homogeneity of variance and Welch ANOVA employed when variances were unequal. Pearson correlation coefficients, and Spearman rank correlations, for measured traits within and between environments, respectively, were calculated using SAS PROC CORR. For the SC NILs and subNILs, estimations of heritability (h^2^) were derived from the ANOVA, and two-way analyses of variance were conducted to examine genotype × environment interactions as described in Eduardo et al. (2007) using PROC GLM. Restricted maximum likelihood method (REML) estimates of the variance components were calculated using SAS PROC VARCOMP where the linear model was completely random.

### Genotyping and QTL analysis

The NIL collection was genotyped with 768 SNPs with the Illumina GoldenGate assay (Illumina, San Diego, CA) (Esteras et al., 2013) as described previously (Argyris et al., 2015a; Table S1). Four additional NILs (SC5-1, SC8-1, SC8-3, and SC12-1) not included in the initial genotyping were later genotyped with a subset of 307 of these SNPs as part of the work described in Argyris et al. (2015b). The size and location of SC introgressions in the PS genetic background were defined using the physical positions of the SNPs in the melon genome according to melon pseudomolecule version 3.5.1 (http://www.melonomics.net/files/Genome/Melon_genome_v3.5.1/) and then translating their positions to the genetic map developed in Argyris et al. (2015b). Genes involved in the sucrose metabolic pathway annotated from the full genome sequence of melon (Garcia-Mas et al., 2012) were also positioned on the map according to their physical locations to identify potential QTL/candidate gene collocations.

For the PS × TRI F_2_ population, a genetic map of 128 SNPs evenly distributed through the genome was obtained as described elsewhere (Diaz et al., 2014). Briefly, selected SNP markers were genotyped with Illumina Veracode (at Centre for Genomic Regulation, Barcelona, Spain) and Sequenom MassArray iPLEX (at Servicio de Investigaciones Biomédicas, Unidad Central de Investigación, University of Valencia, Valencia, Spain). Genetic mapping was performed with MAPMAKER 3.0 (Lander et al., 1987). QTL analysis was carried out for each experiment (VAL11, ZA11, ZA12) using Windows QTL Cartographer 2.5 (Wang et al., 2012) with the composite interval mapping (CIM) (Zeng, 1993) procedure. The LOD score threshold for a significant level *p* < 0.05 was obtained for each trait by a permutation test with 1,000 resamplings, QTL with LOD scores higher than 2.5 but that were declared significant in at least one location after the permutation test are also reported.

Backcross plants (BC1 and BC2 in VAL10 and VAL12 trials) were genotyped with markers associated to sugar content according to F_2_ analysis and a sample of markers located in background genomic regions using the previous Sequenom assay. Heterozygous and PS marker class means were compared with a *t*-test. TRI5-1 was genotyped with the previously described Goldengate array and additional downstream markers that established an introgression size of 26 Mb from the beginning of the chromosome to maker CMPSNP690 (Table S1).

The effects of introgressions on SC NILs and TRI5-1 were studied by calculating the percentage change in a trait by comparing the mean trait value of each NIL against PS as control and then determining significance using the Dunnett's contrast with Type-I error α ≤ 0.05 (Dunnett, 1955). The names of QTL were assigned based on the trait (e.g., *ssc*), followed by a “*q*” for QTL, an abbreviation of the line in which it was detected (e.g., “*sc*” for “Songwhan Charmi” or “*t*” for “Trigonus”), then by a number indicating the chromosome and order of detection (e.g., *5.1*), and then the location abbreviation (e.g., CDM) and the year of the trial in that location.

### SC subNIL development and genotyping

To develop SC sub-NILs, NIL SC5-1 was first crossed with PS to produce F_1_ plants which were genotyped with SNPs selected using the SUPER pipeline (Sanseverino et al., 2015) as described in Argyris et al. (2015b). Based on the size of the SC introgression contained in SC5-1 as determined from the results of NIL genotyping with the GoldenGate assay, a panel of 48 SNPs was chosen to cover the genome interval of 2.9 megabases (Mb) from a physical position on chromosome 5 at 4.89 Mb (the end of CM3.5.1_scaffold00022) to 1.97 Mb (Table S2). This included the QTL and a flanking region with SNPs spaced at approximately 61 kilobase (kb) intervals. Five of these consistently failed to amplify, or were not unequivocal in the parental genotype, so were discarded. This left a panel of 43 functioning SNPs for analyses. To delimit the size of the SC5-1 introgression, the F_1_ plants were genotyped first with the SNP panel by competitive allele-specific polymerase chain reaction (PCR) and KASPar chemistry (KBioscience Ltd., Hoddesdon, UK) using the Fluidigm (Fluidigm Corp., South San Francisco, CA) nanofluidic 48.48 dynamic array (Wang et al., 2009) according to Maughan et al. (2012). Then, three of the 43 SNPs genotyped using KASPar and flanking the ends of the introgression (sca00022_3549755, located at 2,923,752 basepairs (bp) and sca00022_5424269 located at 4,891,016 bp) and another located inside the QTL interval (sca00022_4484619 located at 3,951,366 bp) (Table S2) were chosen for genotyping the F_2_ and subsequent generations with TaqMan® chemistry (ThermoFisher Scientific, Waltham, MA) using the SNP genotyping in Universal Master Mix (Applied Biosystems, Foster City, CA). The F_1_ plants were self-pollinated and F_2_ seeds collected from mature fruits and maintained separately. The flanking and internal markers plus the 43 SNP panel were employed over the course of three additional generations to develop F_4_ subNILs.

### SC subNIL phenotyping

Cultivation and phenotyping of the F_2_ and F_3_ subNILs in CDM was performed under the same conditions as described above for the NILs. DNA extractions were performed using the CTAB method (Doyle and Doyle, 1990) by taking samples at the two-leaf stage from seedlings. In March 2013, 600 F_2_ plants were planted in trays and screened with the three flanking markers to identify recombinant lines. Eighty plants were subsequently transferred to the greenhouse and genotyped with the 43 SNP panel. Fruits from 57 F_2_ lines were phenotyped and QTL cartographer _V_2.5 (Wang et al., 2012) was employed to perform a single marker regression analysis. Following harvest, 48 F_3_ seeds derived from each of 16 F_2_ lines were sown immediately and the genotyping process repeated with flanking markers and the SNP panel to identify lines fixed for SC alleles in defined intervals. Three or four plants of each line were transferred to the greenhouse and self-pollinated. The F_4_ seeds were harvested from the fixed lines.

Two experiments were performed in 2014 by cultivating the F_4_ subNILs in CDM14 and VAL14. Ten plants of each subNIL and PS, plus 5 plants of SC5-1, were grown in a greenhouse in a completely randomized design. Mature fruits were evaluated for SSC and soluble sugar content as described above. Following the identification of *sscqsc5.1* and *sucqsc5.1* in the F_4_ SC subNILs, six additional SNPs spanning 60 kb and located between sca00022_3650228 and CMPSNP437_3732580 (Table S2) were selected and genotyped as described above to further fine-map the QTL.

For the lines showing significant effects compared to the PS control according to Dunnett's contrast, a QTL was considered to be within the genomic region covered by the line. If multiple lines showed the effect, the QTL was considered to be located within the chromosomal region shared by the subNILs.

### Candidate genes variants identification in parental lines

Resequencing data of the parental lines of the populations (PS, SC, TRI), as well as other cultivars and accessions representing both ssp. *melo* (“Védrantais” (VED) group *cantalupensis*), and ssp. *agrestis* (“Calcuta” (CAL) group *momordica* accession PI 124112 and “Cabo Verde” (CV) accession C-836, a wild type) (Sanseverino et al., 2015) were explored to identify variations in the three candidates genes MELO3C014519, MELO3C014521, and MELO3C014522. The PS and VED cultivars are sweet, high sugar accumulating lines, while SC, TRI, CAL, and CV are non-sweet, with low sugar content (Stepansky et al., 1999). As the melon genome sequence is assembled from the double-haploid line DHL92 containing both PS and SC introgressions, SC was established as the reference genome in this interval (Garcia-Mas et al., 2012). Using a new assembly and annotation of the genome (Garcia-Mas et al., unpublished), the SUPER-W pipeline (Sanseverino et al., 2015) was used for the variant calling procedure and vcftools (Danecek et al., 2011) and in house script for a post-filtering process. This allowed selecting high quality variants with a genotype depth >5 and genotpe quality >30. In addition, since a high rate of homozygous variants are expected in melon, heterozygous variants were discarded. SnpEff v4 (Cingolani et al., 2012) was used for imputing the gene region in which the variants fall (promoter, intron, exon, UTRs) and the putative impact of the variants on protein functionality. Subsequently analyses on exonic SNPs and INDELs were performed in order to estimate the deleterious degree of the variants. To do this, a combination of different prediction web tools was exploited, including PROVEAN (Protein Variation Effect Analyzer) (http://provean.jcvi.org/index.php), SIFT (http://sift.jcvi.org/), SNAP2 (https://rostlab.org/services/snap2web/), and Hope (http://www.cmbi.ru.nl/hope/method/). iTASSER (Iterative Threading ASSEmbly Refinement) (http://zhanglab.ccmb.med.umich.edu/I-TASSER/) (Zhang, 2008) was used to predict the conformational structures and protein-ligand binding sites based on sequence variants. The GSDS v2 software (Hu et al., 2015) was used to design gene structure of the candidate genes.

### RNA isolation and qPCR expression analyses

To measure expression of candidate genes MELO3C014519, MELO3C014521, and MELO3C014522 in the subNILs, qPCR analyses were performed as in Saladie et al. (2015) with the following modifications: Total RNA from five different biological replicates for each of four SC subNILs (two high sugar and two low sugar lines), PS and SC5-1 was isolated from mesocarp tissue of each replicate from a 100 mg ± 2 mg sample of previously frozen and ground fruit pulp tissue using TriZOL® reagent (Ambion®, Life Technologies, Inc.) following the manufacturer's instructions. RNA was treated with RNAse free TURBO-DNase I (Turbo-DNA-*free*™ Kit; Applied Biosystems, Ambion®, USA) for 30 min at 37 C, before use as a template for cDNA synthesis. RNA quality was assessed by gel electrophoresis, quantified on a Nanodrop ND-1000 (NanoDrop® Technologies, Wilmington, Delaware), and reversed transcribed into cDNA from 500 ng of total RNA with an oligo(dT)_20_ primer and a SuperScript™ III Reverse Transcriptase kit (Invitrogen, Carlsbad, CA) according to the manufacturer's instructions.

Expression analysis was performed on a LightCycler® 480 Real-Time PCR System using SYBR® Green I Mix (Roche Applied Science, USA). The relative amounts of specific transcripts were determined using cyclophilin (*CmCYP7*) as a reference gene, as in previous experiments (Mascarell-Creus et al., 2009; Saladie et al., 2015). Primers for amplification of target and reference genes were designed with Primer3 software (http://primer3.wi.mit.edu/) (Table S3). To maximize efficiency of qPCR reactions, primers pairs were checked for the presence of secondary structures with NetPrimer (http://www.premierbiosoft.com/netprimer/) and redesigned if necessary. Calculation of intra-assay variation, primer efficiencies, specificity of the PCR amplification, and the presence of genomic contamination were as described previously. The relative expression of target genes was calculated using Cp values calculated by LC480 software. All statistical calculations were performed using ΔCp values, as this parameter followed a normal distribution as assessed by the Kolmogorov-Smirnov test. Data were transformed to a log_2_ scale.

## Results

### Phenotypic variation for sugars and organic acids in SC NILs and TRI-derived populations

Fruits of SC were not included in the VAL trial so comparisons were not possible, but in CDM and CAM, SC had lower levels of SSC, sucrose, and citrate, and higher levels of malate relative to fruits of PS. Glucose and fructose contents were similar for both genotypes. Mean trait values of the NIL population were closer to the mean of PS in all locations except for significantly lower SSC and sucrose values in CAM.

Mean SSC of the NILs varied from 9.8 to 10.5 Brix (°Bx) in each location and showed a wide range, especially in VAL11 (Table 1). The mean SUC contents varied from 37.5 to 44.2 mg g^−1^ fresh weight (FW) in each location, with the highest values and widest range in CDM (up to 73.5 mg g^−1^), compared to lower values in VAL11 and CAM. For GLU, means of the NILs ranged from 13.1 to 19.1 and for FRU from 10 to 16.6 mg g^−1^ FW. For both, NIL means were similar in CDM and CAM, but lower in VAL11. Mean values for MAL were 60x greater in CDM compared to CAM, and was not collected from VAL11. Compared to PS, means of the NILs were similar for all of the traits.

**Table 1:** Soluble solids content (SSC) (°Bx), sucrose (SUC), fructose (FRU), glucose (GLU), citrate (CIT), and malate (MAL) content (mg g^−1^ fresh weight) of SC NILs, PS, SC, and TRI F2 and BC populations.

**Trait**	**Population**	**Location^a^**	**NIL**	**Range**	**PS^b^**	**SC**
SSC	SC NIL	CDM (47)	10.2 ± 1.5	7.9 – 11.9	10.8 ± 1.1	6.9 ± 1.4
		CAM (37)	9.8 ± 1.4	7.6 – 11.6	11.9 ± 1.2	8.2 ± 0.5
		VAL11 (49)	10.5 ± 1.7	6.3 – 13	10.6 ± 0.4	–
	TRI F2	VAL11 (113)	6.7 ± 1.5	3.8 – 9.7		
		ZA11 (113)	5.2 ± 1.3	2.6 – 9.8		
		ZA12 (113)	7.5 ± 1.2	5.2 – 11.6		
	TRI BC	VAL10	6.6 ± 2.4	4.1 – 10.6		
		VAL12	9.2 ± 2.0	5.8 – 14.3		
SUC	SC NIL	CDM (47)	44.2 ± 18.7	20.7 – 73.5	43.9 ± 11	15.1 ± 11.2
		CAM (37)	37.5 ± 12.4	21.4 – 60.6	48 ± 8.6	26.0 ± 7.2
		VAL11 (49)	41.1 ± 13.2	13.8 – 58.5	43.1 ± 14.3	–
	TRI F2	ZA11 (113)	17.8 ± 11.3	2.5 – 51.8		
	TRI BC	VAL10	31.9 ± 26.1	1.1 – 124.5		
		VAL12	31.3 ± 14.1	9.3 – 70.9		
GLU	SC NIL	CDM (47)	18.3 ± 5.5	13.1 – 28.7	18.1 ± 6.9	16.3 ± 3.3
		CAM (37)	19.1 ± 4.5	8.9 – 26.1	17.9 ± 2.6	15.1 ± 2.4
		VAL11 (49)	13.1 ± 2.6	8.0 – 16.8	11.7 ± 1.9	–
	TRI F2	ZA11 (113)	17.9 ± 4.3	7.7 – 28.9		
	TRI BC	VAL10	24.6 ± 6.6	7.4 – 49.2		
		VAL12	17.3 ± 4.5	3.7 – 25.9		
FRU	SC NIL	CDM (47)	16.6 ± 4.2	12.7 – 21.3	16.1 ± 2.8	14.6 ± 4.0
		CAM (37)	13.8 ± 2.7	10.2 – 18.6	14.2 ± 2.5	12.0 ± 1.8
		VAL11 (49)	10 ± 1.9	7.3 – 12.9	8.4 ± 1.7	–
	TRI F2	ZA11 (113)	11.2 ± 3.6	0.7 – 21.2		
	TRI BC	VAL12	13.5 ± 3.1	6.5 – 18.4		
CIT	SC NIL	CDM (47)	2.5 ± 1.2	0.6 – 4.4	2.7 ± 0.9	
		CAM (37)	2.8 ± 1.0	1.8 – 4.7	3.7 ± 1.2	1.1 ± 0.5
		VAL11 (49)	1.8 ± 0.6	1.0 – 2.3	1.7 ± 0.2	1 ± 0.4
	TRI F2	ZA11 (113)	6.5 ± 1.7	1.9 – 11.4		–
	TRI BC	VAL12	3.9 ± 1.2	0.7 – 6.8		
MAL	SC NIL	CDM (47)	0.6 ± 0.4	0.14 – 1.8	0.5 ± 0.5	
		CAM (37)	0.01 ± 0.005	0.01 – 0.03	0.01	0.7 ± 0.8
	TRI F2	ZA11 (113)	0.9 ± 0.5	0.1 – 3.2		0.1 ± 0.03
	TRI BC	VAL12	0.51 ± 0.7	0.13 – 3.3		

a *In CAM, 12 NILs did not produce fruit (SC1-4, 3-5, 5-3, 5-5, 6-2, 6-6, 7-4, 8-5, 9-1, 9-2, 9-3, and 12-3) and were not analyzed*.

b *Data for PS was not available for TRI-derived populations*.

*Means ± SD. Numbers in parentheses are n number of lines evaluated in each location*.

For all the TRI-derived lines, SSC and SUC contents were lower, GLU and FRU similar, and CIT and MAL higher than in the SC NILs. Fruits from the TRI- F_2_ population showed moderate SSC values (mean between 5.2 and 7.5 °Bx in all three locations (VAL11, ZA11 and ZA12) but with a wide range (2.57–11.6 °Bx). The mean SUC value (17.8 mg g^−1^ FW) in ZA11 was very low, while GLU and FRU values ranged from 7.7 to 29 and 0.7 to 21.2 mg g^−1^ FW, respectively. The mean CIT and MAL values were 6.5 and 0.9 mg g^−1^ FW, respectively. Compared to the F_2_ lines, mean SSC for the TRI BC populations were similar in VAL10 but higher in VAL12, with a mean of 9.2 °Bx. The SUC concentrations were significantly higher in both locations, with a very wide range. GLU and FRU were higher in VAL10, and similar in VAL12. In contrast, CIT and MAL were both higher in the BC lines compared to the F_2_.

For the SC NILs, the Welch ANOVA indicated a highly significant genetic (NIL), environment (LOC), and interaction of genotype × environment (NIL^*^LOC) effect for all traits in every location (*P* ≤ 0.001) (Table S4). The contribution of the NIL component to total variation of the studied traits was very low, except for SSC where it contributed up to 18% (Table S5). Contribution of LOC to total variance for SSC and SUC was also low (4% for both locations), whereas NIL^*^LOC interactions were higher (33 and 23%, respectively). The LOC effect was higher for GLU, FRU, MAL, and CIT, ranging from 54 to 22%, with CIT and MAL also having a strong NIL^*^LOC effect (25 and 26%, respectively). Heritability estimates derived from the ANOVA were highest across locations for SSC (range, 0.32–0.61) and lowest for FRU. Heritability for SUC ranged from 0.2 to 0.51.

### Correlation analyses

The strongest correlations for all traits were observed between CAM and VAL11, especially for SSC (0.49) and SUC (0.5) while correlations for the same traits between CDM and both CAM and VAL11 were weaker (Table S6). There was almost no correlation for SSC and a negative one for SUC among the CDM and VAL locations (0.03 and –0.27, respectively) reflective of the strong NIL^*^LOC effects for the two traits and that the ranking of the NILs was distinct in each environment.

Within locations, highly significant positive correlations were observed between GLU and FRU, ranging from 0.48 to 0.92. GLU and FRU also showed moderate to strong negative correlations with SSC and SUC in each environment. SUC content in melon fruits was moderately correlated to SSC in both VAL11 and CAM (0.56 and 0.50, respectively) but very weakly in CDM (0.18). MAL and CIT showed a strong negative correlation in CDM and were not correlated, or only weakly so. Reflecting these trends in each environment, correlations for data combined across environments were similar, with a moderate overall correlation between SSC and SUC (0.41).

### QTL analysis of SC NILs

Results of the SC NIL genotyping were described previously (Argyris et al., 2015a) and more detail on positions and sizes of SC introgressions in the NILs is provided in Table S1. Overall, 24 SC NILs showed a significant difference from the PS control with Dunnett's test (*p* < 0.05) for one or more of the measured phenotypic traits in one location, thus indicating the presence of putative QTL within the introgressions. With this criteria, 55 QTL were detected (Table 2). The phenotypic values affected by putative QTL ranged from up to a 252% increase (*mal8.1*), to 78% decrease (*cit8.1)* in the trait relative to PS. With one exception (*sscqsc3.5_VAL11*), SC alleles decreased trait values for SSC, SUC, and CIT, and except for *fruqsc6.4, fruqsc8.2*, and *gluqsc8.2*, generally increased values for GLU, FRU, and MAL content.

**Table 2:** QTL detected in SC NILs for soluble solids content (SSC) (°Bx), sucrose (SUC), fructose (FRU), glucose (GLU), citrate (CIT), and malate (MAL) content (mg g^−1^ fresh weight) and comparison to trait values in PS.

**QTL name^a^**	**Chr**	**Start interval (Mb)^b^**	**End interval (Mb)**	**Trait value^c^**		**Change relative to PS (%)**
				**NIL**	**PS**	
*sscqsc1.5_CAM*	1	33.3	34.7^*^	9.1 ± 1.4	11.9 ± 1.2	−23.3
*sscqsc2.1_CAM*	2	0	1.4	8.9 ± 1.7	11.9 ± 1.2	−25.2
*sucqsc2.1_CAM*	2			30.3 ± 5.9	48 ± 8.7	−36.9
*sscqsc3.2_CAM*	3	2.8	15.4	8.8 ± 2	11.9 ± 1.2	−25.8
*sucqsc3.2_CAM*	3			28.3 ± 16.7	48 ± 8.7	−41
*sscqsc3.5_VAL11*	3	21.1	27	13 ± 1.2	10.6 ± 0.4	23
*sscqsc4.1_CAM*	4	3.9	14.3	8.2 ± 0.8	11.9 ± 1.2	−31.4
*sscqsc4.1_VAL11*	4			7.4 ± 1.0	10.6 ± 0.4	−30.2
*sucqsc4.1_CAM*	4			25.8 ± 5.1	48 ± 8.7	−46.2
*sucqsc4.1_VAL11*	4			25.7 ± 8.3	48 ± 4.0	−40.4
*gluqsc4.1_VAL11*	4			14.9 ± 2.5	11.1 ± 0.8	27.6
*sscqsc4.3_CAM*	4	3.9	27.4	9.0 ± 0.5	11.9 ± 1.2	−24.7
*sscqsc5.1_CAM*	5	0.3	1.7	8.7 ± 0.7	11.9 ± 1.2	−26.9
*sscqsc5.1_CDM*	5			7.9 ± 1.6	10.5 ± 1.3	−27.3
*sscqsc5.1_VAL11*	5			6.9 ± 1.2	10.6 ± 0.4	−34.7
*sucqsc5.1_VAL11*	5			14 ± 6.4	48 ± 4.0	−67.5
*fruqsc5.1_VAL11*	5			12.4 ± 1.2	7.9 ± 0.7	47.1
*gluqsc5.1_VAL11*	5			16.4 ± 1.3	11.1 ± 0.8	40.3
*sscqsc5.2_CAM*	5	0.3	5.9^*^	7.6 ± 0.9	11.9 ± 1.2	−36.1
*sscqsc5.2_CDM*	5			8.4 ± 1.4	10.5 ± 1.3	−21.9
*sucqsc5.2_CAM*	5			21.4 ± 9.8	48 ± 8.7	−55.4
*sucqsc5.4_CAM*	5	27.3	28.1	28.6 ± 8.7		−40.4
*fruqsc6.2_VAL11*	6	0.5	5.3	11.4 ± 1.2	7.9 ± 0.7	35.7
*gluqsc6.2_VAL11*	6			15.6 ± 2.0	11.1 ± 0.8	33.5
*fruqsc6.4_CAM*	6	0.5	30.6	10.2 ± 1.0	14.3 ± 2.5	−27.9
*sscqsc6.4_CAM*	6			8.5 ± 1.3	11.9 ± 1.2	−28.3
*fruqsc6.6_VAL11*	6	33.1	35.6	10.8 ± 1.0	7.9 ± 0.7	28.2
*sscqsc7.1_CAM*	7	2.4	6.6	10.3 ± 0.3	11.9 ± 1.2	−13.5
*sscqsc7.3_CAM*	7	13	22.3	8.5 ± 0.5	11.9 ± 1.2	−28.3
*sucqsc7.3_CAM*	7			30.6 ± 5.6	48 ± 8.7	−36.3
*fruqsc7.3_VAL11*	7			11.8 ± 1.3	7.9 ± 0.7	40.8
*gluqsc7.3_VAL11*	7			15.6 ± 1.6	11.1 ± 0.8	33.6
*citqsc7.3_CAM*	7			2 ± 0.7	3.7 ± 1.2	−45.2
*sscqsc7.4_VAL11*	7	13	25.5	8.2 ± 0.7	10.6 ± 0.4	−23
*sucqsc7.4_VAL11*	7			25.6 ± 7.4	48 ± 4.0	−40.7
*fruqsc7.4_VAL11*	7			12.3 ± 2.2	7.9 ± 0.7	46.7
*gluqsc7.4_VAL11*	7			16.8 ± 2.9	11.1 ± 0.8	43.3
*citqsc8.1_CDM*	8	0.3	5.3	0.6 ± 0.3	2.7 ± 0.9	−78
*malqsc8.1_CDM*	8			1.8 ± 0.5	0.5 ± 0.5	252
*citqsc8.2_CDM*	8	1.6	14.9^*^	0.7 ± 0.6	2.7 ± 0.9	−74.2
*malqsc8.2_CDM*	8			1.8 ± 0.4	0.5 ± 0.5	245.7
*fruqsc8.2_CAM*	8			10.6 ± 1.7	14.3 ± 2.5	−25.5
*gluqsc8.2_CAM*	8			8.9 ± 3.5	17.9 ± 2.6	−50.5
*sucqsc8.3_CAM*	8	6.1	29.5	33.4 ± 10.9	48 ± 8.7	−30.4
*fruqsc8.4_VAL11*	8	19.3	32.5	12.9 ± 3.7	7.9 ± 0.7	53.8
*gluqsc8.4_VAL11*	8			15.2 ± 3.8	11.1 ± 0.8	29.8
*gluqsc9.1_VAL11*	9	0.1	4.2^*^	14.2 ± 1.5		21.1
*citqsc10.3_CAM*	10	3.4	4.7	2.3 ± 0.8	3.7 ± 1.2	−37.6
*malqsc11.1_CAM*	11	2.5	3.7	0.03 ± 0.01	0.01	184.8
*sscqsc11.2_CAM*	11	1.2	25.4	7.7 ± 1.2	11.9 ± 1.2	−35.7
*sucqsc11.2_CAM*	11			22.0 ± 5.7	48 ± 8.7	−54.2
*fruqsc11.2_VAL11*	11			12.1 ± 2.4	7.9 ± 0.7	44.4
*gluqsc11.2_VAL11*	11			16.7 ± 3.1	11.1 ± 0.8	42.3
*gluqsc11.4_CDM*	11	17.6	30.7	28.7 ± 2.4	18.2 ± 6.9	58.3
*sscqsc12.1_CAM*	12	5.1	23.6	8.5 ± 2.3	11.9 ± 1.2	−29.4

a *Names of QTL are assigned based on the measured trait: SSC, SUC, FRU, GLU, CIT, and MAL, followed by a “q” designating QTL, an abbreviation of its origin as Songwhan Charmi (sc), then by a number indicating the NIL in which it was detected, and then the location and year of trial (see text)*.

b *Start and end intervals in megabases (Mb) on target chromosome (Chr) pseudomolecule according to genome version 3.5.1*.

c *Units for SSC are expressed in °Bx; for SUC, FRU, GLU, CIT, and MAL, trait values are expressed in mg g^−1^ fresh weight*.

* *Includes portion of the interval in heterozygosis*.

*Trait means ± SD*.

### QTL affecting SSC and soluble sugars in SC NILs

Clusters containing “stable” QTL, in which SSC plus soluble sugars content were affected in at least 2 locations, were detected on chromosomes 4, 5, and 7. Since pairs of NILs sharing a part of their SC introgression were detected for the same traits at these genome positions, they likely represent single QTL.

On chromosome 4, a cluster of six QTL was detected for SSC, SUC, and GLU, in SC4-1 and SC4-3, which shared a large SC interval of 10.4 Mb from 3.9 to 14.3 Mb (Table S1). The two QTL detected in SC4-1 reduced SSC by 31–30% in CAM and VAL11, and another detected in SC4-3 in CAM reduced SSC by 25%. Two QTL were detected in SC4-1 that reduced SUC content by 46 and 40% in CAM and VAL11, respectively, while increasing GLU content only in VAL11.

**Figure 1:**
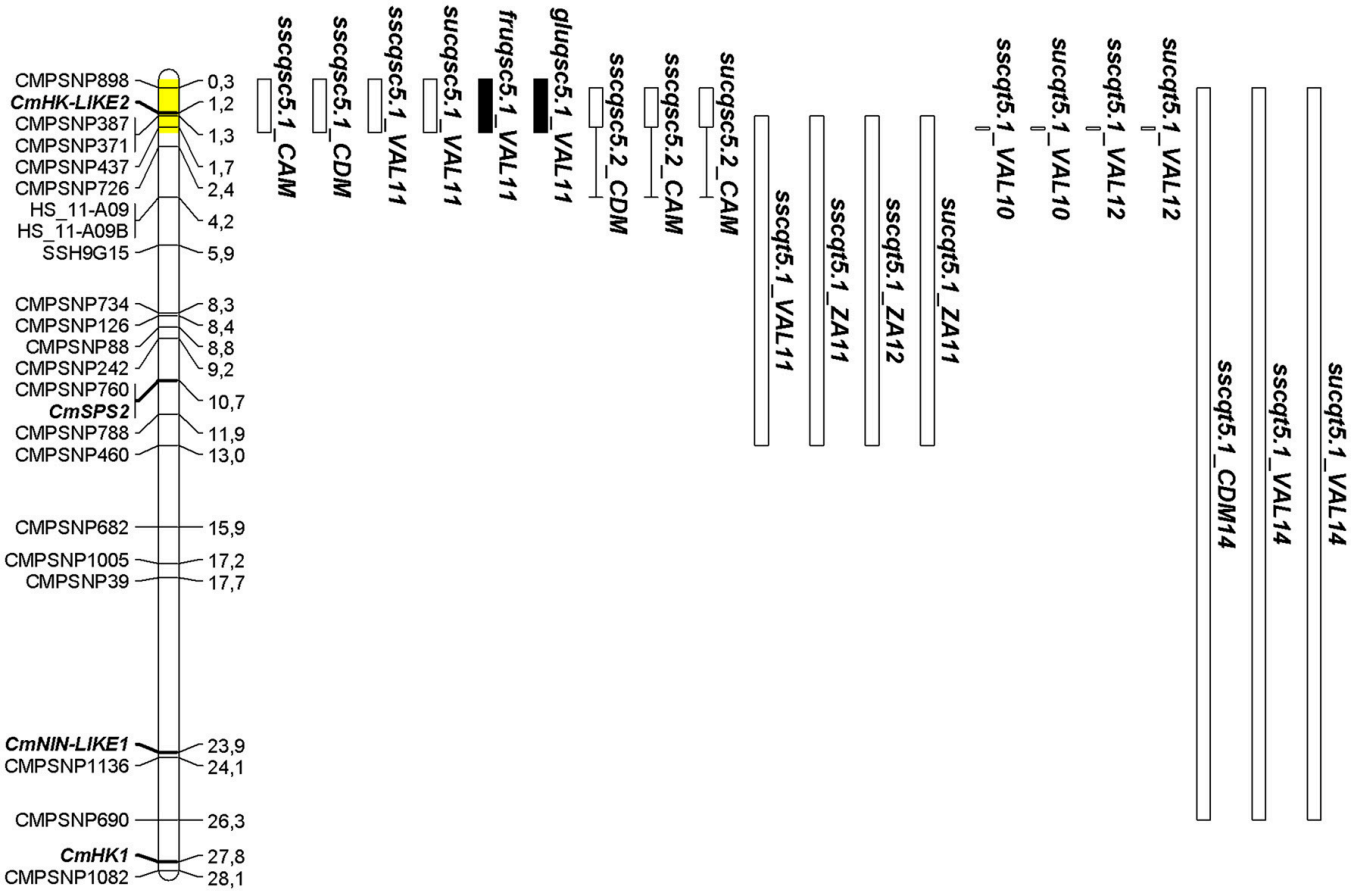
Positions of QTL detected in SC5-1 and SC5-2 within a 1.88 Mb interval on chromosome 5 (shaded in yellow) colocating with those detected in TRI-derived lines to a portion of the same interval. Locations of SNPs and sucrose metabolism genes (bold italics) are given in megabases (Mb). QTL nomenclature for traits is given as in Tables 2, 3. QTL intervals defined by black and white bars represent an increase or decrease in the trait value, respectively. The length of the bar represents the physical size of the SC introgression while areas of heterozygosity are indicated by bracketed line. For the TRI-derived F_2_ (VAL11, ZA11, ZA12) and BC (VAL10, VAL12) lines, length of bar represents the 1-LOD QTL confidence interval or SNP position showing the most significant linkage, respectively. For TRI5-1, length of QTL bars for *sscqt5.1_CDM14, sscqt5.1_VAL14*, and *sucqt5.1_VAL14* represents the physical size of the TRI introgression.

On chromosome 5, a cluster of nine QTL was detected for SSC, SUC, FRU, and GLU in SC5-1 and SC5-2 (Figure 1). The NILs shared an SC introgression of 1.7 Mb beginning at the top of chromosome 5 at SNP CMPSNP898 and ending at CMPSNP437. The interval was extended to 1.88 Mb following the results of genotyping with the SNP panel (Table S2) as described below. SC5-2 also contained a second heterozygous interval of 1.71 Mb from 4.19 Mb (SNP HS_11-A09) to 5.9 Mb (SNP SSH9G15) (Table S1). QTL detected in SC5-1 individually reduced SSC between 35 and 27% in all three locations, and by a combined average of 25%. SC5-1 also showed a 67% reduction in SUC, and a 40 and 47% increase in GLU and FRU content respectively, in VAL11. Two QTL detected in SC5-2 in CDM and CAM reduced SSC by 22–36%, while a third identified in CAM reduced SUC content by 55%.

A cluster of nine QTL was detected for five traits on chromosome 7 in SC7-3 and SC7-4. Five QTL were detected in SC7-3; one each reducing SSC and SUC by 28 and 36%, respectively, and a third increasing CIT in CAM. Two other increased GLU by 41% and FRU by 34% in VAL11. SC7-4 reduced SSC by 23% and sucrose by 41% in VAL11, while increasing GLU and FRU, by 43 and 47%, respectively. The NILs shared a common interval of 9.3 Mb from 12.9 to 22.3 Mb (Table S1), so may represent single QTL for SSC, sucrose, glucose and fructose. However, as SC7-2 was not detected as being significantly different for any of the measured traits despite also sharing the common interval with SC7-3 and SC7-4, it is possible that the QTL in the latter two lines were distinct.

### QTL for organic acids on chromosome 8 in SC NILs

Two NILs detected in CDM, SC8-1 and SC8-2, showed both significantly reduced citrate (0.59 and 0.70 mg g^−1^, corresponding to 78 and 74% reduction, respectively) and a highly significant increase in malate content (1.76 and 1.73 mg g^−1^, corresponding to 252 and 246% increase). The two lines shared at least a 1.23 Mb homozygous introgression on chromosome 8 from 4.1 to 5.9 Mb, and an additional area of shared heterozygosity which also overlapped with QTL for reduced fructose and glucose detected in SC8-2 in CAM.

Outside of the clusters, seven other NILs decreased SSC in a single location only. One NIL (SC3-5) showed significantly higher mean SSC (13.0), a 23% increase compared to PS. Five NILs showed significantly reduced SUC content in a single location only, while none were detected for increased SUC content. Six NILs were detected for GLU and FRU, two for CIT, and one for MAL, also all in single locations only.

### QTL analysis in TRI-derived populations

In the TRI-derived populations, 23 QTL were detected (Table 3) most for SSC and SUC. For the F_2_ lines two QTL for SSC with consistent effects in two or more locations were identified in chromosomes 3 and 5. For chromosome 3, two detected in ZA11 and ZA12 reduced SSC, accounting for 8–9% of the phenotypic variation in the trait. On chromosome 5, four QTL detected in 3 different locations in the genome interval from 1.3 to 13 Mb reduced SSC and SUC, accounting for from 11 to 44% of the phenotypic variation. Additional QTL for SSC were identified only in ZA12 and VAL11 on chromosomes 4 and 8, respectively. Sugar and organic acid composition was investigated only in ZA11, with two QTL on chromosomes 8 and 10 for CIT, one in chromosome 3 for FRU, one for MAL on chromosome 2, and another for SUC on chromosome 10 (*sucqt10.1_ZA11)* which increased SUC.

**Table 3:** QTL detected in TRI-derived lines for SSC, soluble sugars SUC, GLU, and FRU, and organic acids (MAL and CIT).

**QTL name^a^**	**Pop**	**Chr**	**Start interval (Mb)**	**End interval (Mb)**	**Trait value**	**LOD**	**Change relative to PS (%)**	**P**	**R^2^**
*malqt2.1_ZA11*	F2	2	0	3.6		8.2			37
*sscqt3.1_ZA11*	F2	3	25.2	27.4		3.5			8
*sscqt3.1_ZA12*	F2	3				4			9
*fruqt3.1_ZA11*	F2	3	3.6	22.4		5.1			16
*sscqt4.1_ZA12*	F2	4	13.9	18.3		4.1			18
*sscqt5.1_VAL11*	F2	5	1.3	11.3		2.8			44
*sscqt5.1_ZA11*	F2	5	1.3	13		3.5			18
*sscqt5.1_ZA12*	F2	5				4.8			11
*sucqt5.1_ZA11*	F2	5				3.6			23
*sscqt8.1_VAL11*	F2	8	1.9	3.2		4.4			21
*citqt8.2_ZA11*	F2	8	6.4	29.5		6			27
*sucqt10.1_ZA11*	F2	10	0.4	2		4.5			54
*citqt10.1_ZA11*	F2	10	4.7	21.1		5.2			26
*sscqt3.1_VAL10*	BC1	3	23.7^*^				−1	0.006	9.2
*sucqt4.1_VAL10*	BC1	4	26.9^*^				−116.8	0	15.6
*sscqt5.1_VAL10*	BC1	5	1.7^*^				−1.1	0.002	11.4
*sucqt5.1_VAL10*	BC1	5	1.7^*^				−84.8	0.009	8
*sscqt12.1_VAL10*	BC1	12	13.9^*^				−0.98	0.005	9.6
*sscqt5.1_VAL12*	BC2	5	1.7^*^				−1.57	0.001	13
*sucqt5.1_VAL12*	BC2	5	1.7^*^				−59.2	0.02	14
*sscqt5.1_CDM14*	IL	5	0.3	26.3	11.6 ± 1.9		−17.1		
*sscqt5.1_VAL14*	IL	5			6.8 ± 0.7		−36.5		
*sucqt5.1_VAL14*	IL	5			31.2 ± 25.6		−44.5		

a Names of QTL are assigned as in Table 1. Origin denoted by (t) from “Trigonus.”

* *Position of marker showing strongest linkage to phenotype according to t-test*.

*Trait means (± SD)*.

In the BC_1_ and BC_2_ populations, QTL for SSC on chromosome 3, 5, and 12 were detected, together with QTL for SUC on chromosomes 4 and 5. Similar to the F_2_ generation, the QTL located on chromosome 5 reduced SSC and SUC in two different years (VAL10 and VAL12). They were strongly linked to SNP CMPSNP437 at 1.7 Mb which was also located within the F_2_ QTL interval.

### Comparison of mapping populations

In comparing the SC and TRI populations, QTL collocated to similar chromosomal intervals for several traits: SSC in chromosomes 3 and 12, SSC and SUC in chromosome 4 and 5, and CIT on chromosome 8 and 10. Among all of them, SSC/SUC QTL on chromosome 5 were the most consistent in both populations, collocating with the QTL cluster identified in the SC NILs (Figure 1). The IL TRI5-1 was therefore constructed to further verify the effects of this QTL in a full PS genetic background. The TRI introgression on chromosome 5 encompassed 26.6 Mb from the start of the chromosome, therefore overlapping with the SC5-1 introgression. Two small additional introgressions on chromosomes 9 and 12 were also present (Table S1). TRI5-1 showed a mean reduction in SSC of 27%, across locations, and a 45% reduction in SUC in VAL14 (Table 3). The QTL contained in this line reducing the traits were denoted as *sscqt5.1_CDM14* and *sscqt5.1_VAL14* and *sucqt5.1_VAL14*.

### Development of subNILs from NIL SC5-1

**Figure 2:**
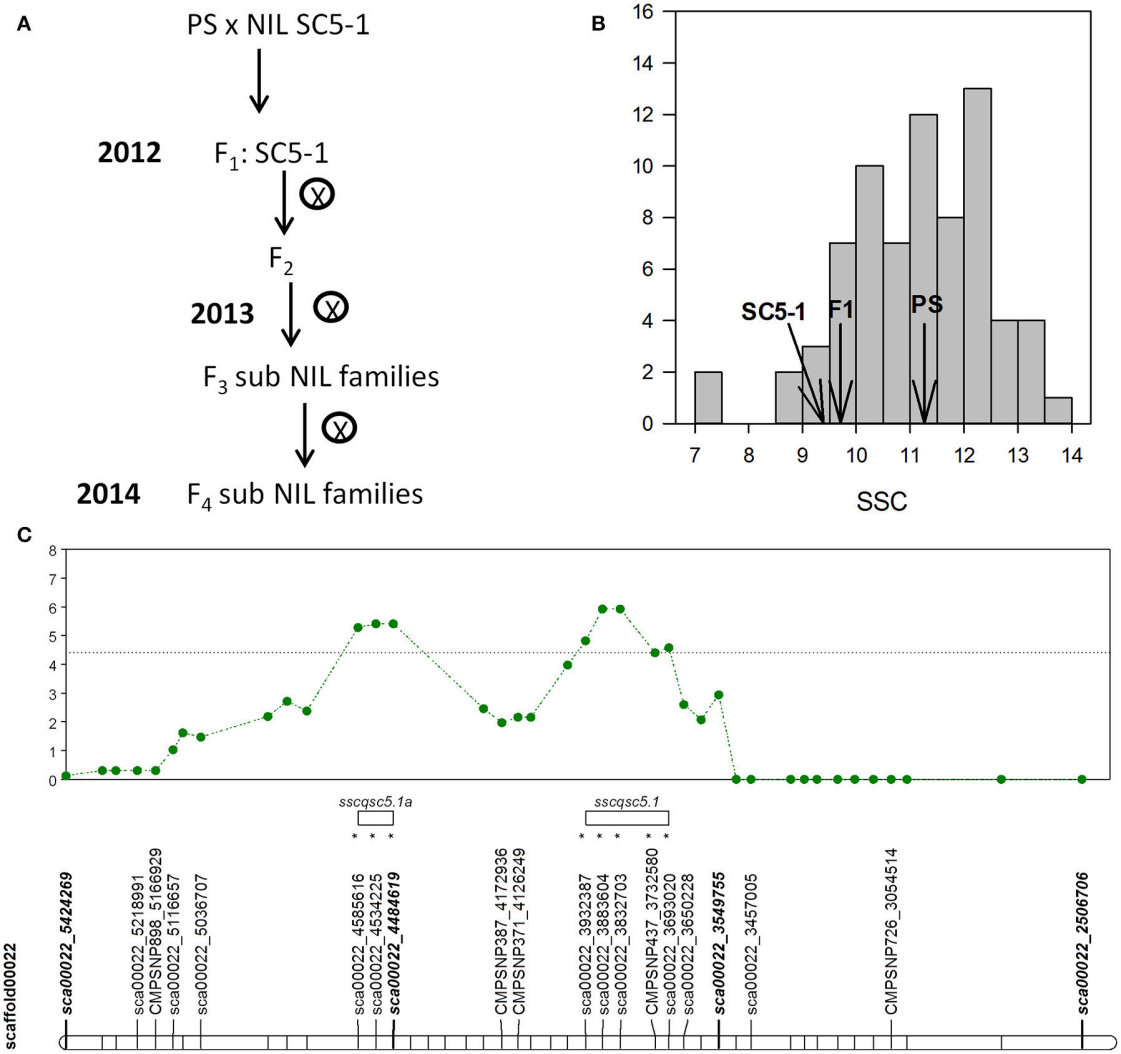
**(A)** Breeding scheme used in the SC subNIL development **(B)** SSC values of melon fruits of the F_2_ SC subNIL population, parental lines, and F_1_ hybrid harvested at 55 DAP. **(C)** Shows single marker QTL analysis of F_2_ population with likelihood ratio (LR) test statistic profile and significance threshold (dotted line) for SSC with SNPs on scaffold CM3.5.1_scaffold00022 (horizontal bar) on chromosome 5. Markers with (^*^) show significant linkage to the QTL at the *p* < 0.05 level. QTL intervals are denoted by open bars.

Based on the consistent location of QTL affecting SSC and SUC in the SC NILs and TRI5-1 (Figure 1), NIL SC5-1 was chosen for fine-mapping through the development and analysis over four generations of new recombinant sub-NILs (Figure 2A). The genotyping of the F_1_ lines PS × SC5-1 cross with a 43 SNP panel indicated that the interval was slightly larger than estimated with the initial genotyping, corresponding to a physical distance of approximately 1.88 Mb from the end of genome scaffold00022 at 4.89 Mb to SNP sca00022_3549755 at 3.02 Mb (Table S2). The recombination breakpoint was located between the latter SNP and sca00022_3457005 at 2.92 Mb. SNPs downstream of 2.92 Mb to SNP sca00022_2506706 at 1.97 Mb were fixed for PS alleles. As the scaffold is negatively oriented in the chromosome 5 pseudomolecule, this interval corresponded to the top of the chromosome in a telomeric region of high recombination (Argyris et al., 2015b) containing approximately 250 genes (http://www.melonomics.net/genome/).

### Phenotyping of subNILs

The SSC values of the 57 plants of the F_2_ population developed from PS × SC5-1 ranged from 7.1 to 13.9 °Bx, with a population mean of 10.8, and parental means of 11.2 and 9.4 °Bx for PS and SC5-1, respectively (Figure 2B). Data for SSC were normally distributed and skewed toward higher values. Single marker regression analysis with 27 of 34 SNPs segregating in the 1.88 Mb QTL interval between sca00022_3549755 and sca00022_5424269, and for which genotyping data was complete, identified significant associations between 8 SNPs contained in two separate likelihood ratio (LR) peaks on chromosome 5, thus indicating linkage of the markers to a QTL (Figure 2C, Table S7). The first putative QTL (*sscqsc5.1*) encompassed 5 SNPs in an interval of 239 kbp, flanked by SNPs sca00022_3932387 at 1,494 kbp and sca00022_3693020 at 1,733 kbp, and accounted for up to 8% of the phenotypic variation. The second putative QTL (*sscqsc5.1a*) encompassed 101 kbp with flanking SNPs sca00022_4585616 at 841 kbp and sca00022_4484619 at 942 kbp, accounting for up to 10% of the phenotypic variation at the maximum LR peak.

**Figure 3:**
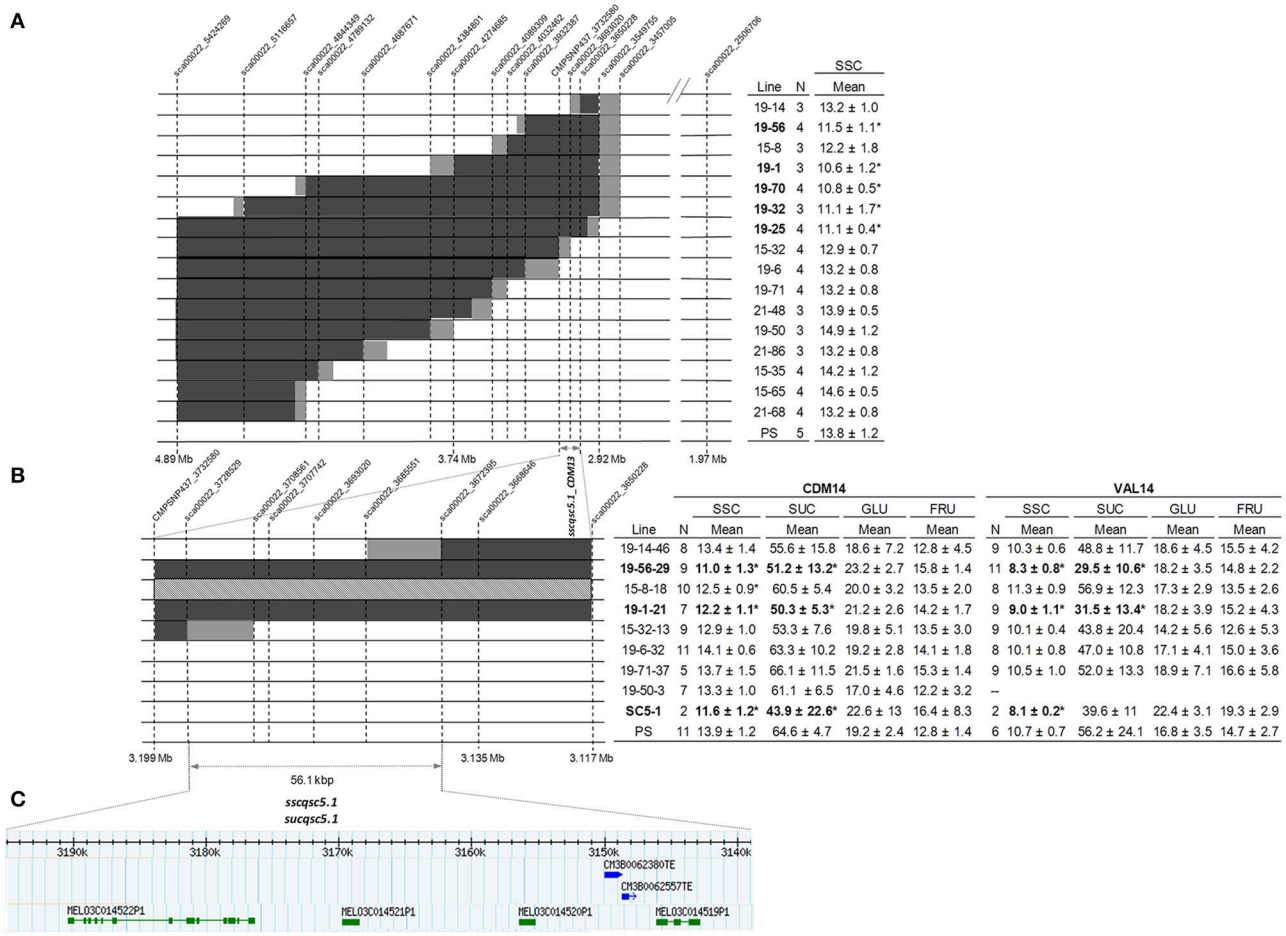
Fine mapping of *sscqsc5.1* and *sucqsc5.1* on genome scaffold CM3.5.1_scaffold00022 on chromosome 5 using the PS × SC5-1 subNILs. Genomic regions containing PS alleles are indicated in white, SC in black, heterozygous in light gray, and regions containing a recombination break point, in dark gray. **(A)** QTL location was narrowed to an 82.4 kbp interval between sca00022_3650228 and CMPSNP437_3732580 in 16 F_3_ subNILs as indicated by significant differences in SSC (^*^) in the table (top right) according to Dunnett's test (*p* < 0.05) compared to PS. Means ± SD. **(B)** Two recombinant lines and an additional 6 SNPs further delimited a 56.1 kbp interval between sca00022_3672395 and sca00022_3728529 as indicated by significant differences in SSC and sucrose in CDM14 (middle table left) and VAL14 (middle table right) in eight F_4_ subNILs, compared to PS. **(C)** Predicted candidate genes (green) and transposons (blue) contained in the QTL interval according to physical position on the genome scaffold.

The F_2_ plants were advanced to the F_3_ generation to produce homozygous lines with introgressions of SC alleles ranging in size from approximately 100 kbp (line 19-14)g to 1, 8 Mb (line 19-25) that together covered the entirety of the original interval contained in SC5-1 (Figure 3A). Between three and four replicates for each family, and five plants of the control (PS), were analyzed for SSC. Mean of 16 the F_3_ families varied between 10.6 and 14.9 °Bx, with PS at 13.8. Five subNILs (19-56, 19-1, 19-70, 19-32, and 19-25) showed significantly reduced SSC compared to the control with Dunnett's test (*p* < 0.05) and were deemed “low” sugar, compared to “high” sugar lines which were not significantly different from the control. The QTL in the low sugar lines (*sscqsc5.1_CDM13*) reduced SSC by an average of 20% and was delimited to a 82.4 kbp genomic interval bounded by SNP markers sca00022_3650228 and CMPSNP437_3732580, and including SNP sca00022_3693020. The latter two SNPs were linked to the 239 kbp QTL interval identified in the F_2_ lines (*sscqsc5.1*; Figure 2C), while none of the five F_3_ subNILs with SC alleles only in the 2nd putative QTL region (*sscqsc5.1a*; 15-32, 19-6, 19-71, 21-48, 19-50) showed significantly reduced SSC. A sixth subNIL (15-8) also shared this interval, but possibly due to the few replicates and high variability in the SSC measurement, was classified as a high sugar line despite having the lowest SSC value within that group.

Mean SSC of eight F_4_ progeny lines derived from a subset of the high and low sugar F_3_ lines ranged from 11 to 14.1 °Bx in CDM14, and from 8.3 to 11.3 in VAL14 (Figure 3B, Table S8). The subNILs 19-1-21 and 19-56-29, progeny of the low sugar F_3_ lines 19-1 and 19-56, were again categorized as low sugar in both locations. The QTL contained in the lines reduced SSC by an average of 17% in CDM (*sscqsc5.1_CDM14*) and 19% in VAL (*sscqsc5.1_VAL14*), and by a combined average of 18%. Similarly, the QTL reduced SUC by an average of 21% in CDM (*sucqsc5.1_CDM14*) and 46% in VAL (*sucqsc5.1_VAL14*), and by a combined average of 34%. Line 15-8-18, which contained the QTL interval in heterozygosis, also showed significantly reduced SSC in CDM14. Since the QTL were detected in both locations, we collectively called them *sscqsc5.1* and *sucqsc5.1*.

The high sugar lines 19-14-46 and 15-32-13 both had informative recombinations flanking the QTL and were genotyped with six additional SNP markers (Table S2). Together they further delimited a 56.1 kbp genome interval from 3,139 to 3,195 Mb on CM3.5.1_scaffold00022 bounded by sca00022_3672395 and sca00022_3728529 (Figure 3C). As these markers were located within the SC introgression in SC5-1 and SC5-2, were linked to 239 kbp QTL interval observed in the F_2_ lines (Figure 2C), and were present in the overlapping SC intervals contained in the low sugar lines in the F_3_ and F_4_ lines, *sscqsc5.1* and *sucqsc5.1* were likely delimited by this region.

### Sequence comparison and expression patterns of candidate genes for *sscqsc5.1* and *sucqsc5.1*

Based on functional annotations in the melon genome database (http://www.melonomics.net/genome/) the QTL interval contained four predicted genes (Table 4): MELO3C014519 is a predicted gene of 3.9 kbp with four exons that encodes a BEL1-like 1 homeodomain protein belonging to the TALE (for three *a*mino acid *l*oop *e*xtension) superclass of transcription factors (Burglin, 1997); MELO3C014520 is most similar to a E3 ubiquitin-protein ligase (PUB22) with a gene size of 1.3 kb and a single exon; MELO3C014521 is a gene of size 2.1 kbp also contained in a single exon and predicted to encode a chloroplastic fantastic four (FAF- like) protein with a domain of unknown function and a transit peptide targeting it to the chloroplast (http://www.uniprot.org/uniprot/Q0V865); Lastly, MELO3C014522 is a larger gene of 14.6 kbp with 13 exons predicted as a serine/threonine-protein phosphatase (BSL2). The interval also contains two predicted transposons (CM3B0062380TE, CM3B0062557TE) adjacent to MELO3C014519.

**Table 4:** Candidate genes contained within the *sscqsc5.1* and *sucqsc5.1* QTL intervals according to the melon genome annotation v 3.5.1.

**Gene ID**	**Gene orientation**	**scaffold position—start**	**scaffold position—**	**PM–start**	**PM–end**	**Size w/introns**	**Function**
MELO3C014519	–	3,143,237	3,147,173	1,746,367	1,750,303	3,936	Similar to BEL1-like homeodomain protein 1 (*Arabidopsis thaliana*) (uniprot_sprot:sp|Q9SJ56|BLH1_ARATH)
MELO3C014520	+	3,156,700	3,157,969	1,735,571	1,736,840	1,269	Similar to E3 ubiquitin-protein ligase PUB22 (*Arabidopsis thaliana*) (uniprot_sprot:sp|Q9SVC6|PUB22_ARATH)
MELO3C014521	–	3,170,295	3,172,387	1,721,153	1,723,245	2,092	Similar to Protein FAF-like, chloroplastic (*Arabidopsis thaliana*) (uniprot_sprot:sp|Q0V865|FAFL_ARATH)
MELO3C014522	+	3,176,506	3,191,115	1,702,425	1,717,034	14,609	Similar to Serine/threonine-protein phosphatase BSL2 (*Arabidopsis thaliana*) (uniprot_sprot:sp|Q9SJF0|BSL2_ARATH)

*Positions and sizes in base pairs (bp) on chromosome (chr) 5 pseudomolecule (PM)*.

Expressed sequence tags (ESTs) were detected in public databases for MELO3C014519 and MELO3C014521 in both developing and mature melon fruit libraries, for MELO3C014522 in mature fruit (http://www.icugi.org/cgi-bin/ICuGI/tool/blast.cgi). Although ESTs were present in callus and root tissue libraries, for MELO3C014520, none were present in fruit libraries. Data from transcriptomic studies, including the parental lines used in developing the NIL and subNIL populations, showed expression of MELO3C014519, MELO3C014521, and MELO3C014522 in developing and mature fruits up to 55 DAP (Saladie et al., 2015; Argyris et al., unpublished). MELO3C104520 was not expressed at any point in fruit development and maturation in these studies, however. Thus, given both the lack of ESTs and absence of expression in fruit, the gene was excluded from further consideration as a potential candidate for *sscqsc5.1*/*sucqsc5.1*.

**Figure 4:**
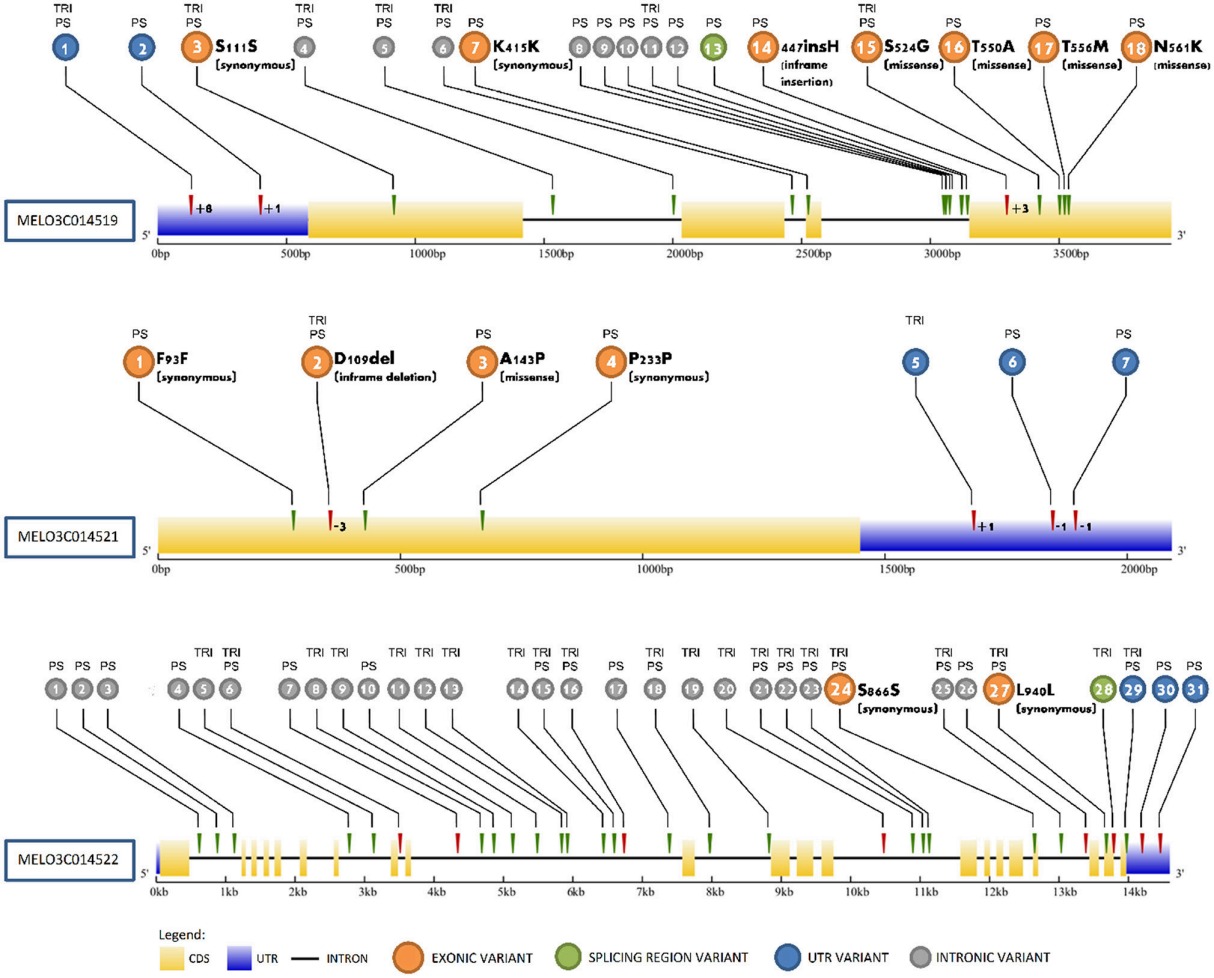
Sequence variations in MELO3C014519, MELO3C014521, and MELO3C014522. Variants are numbered according to Table S9 in colored circles, with alleles carrying the variant compared to the SC as reference genome denoted on top. Gene regions and variant types are noted in the legend.

Analyses of resequencing data between PS, SC, and TRI identified variations in MELO3C014519, MELO3C014521, and MELO3C014522 that differentiated the high and low sugar lines (Figure 4, Table S9). MELO3C014519 harbored 18 variants (15 SNPs and 3 INDELs). Among them, about 11% were found within the 5′- untranslated region (UTR), 39% within exons, 44% within introns and approximately 6% within splicing regions. MELO3C014519 contained 11 variants, including three missense variants (N561K, T556M, T550A) and 1 inframe insertion (D476_H477insH) present only in PS. The same variants were detected in VED, and were absent in the CV and CAL alleles when the analysis was extended to other resequenced lines. There were seven variants in common between PS and TRI which were also present in the other resequenced lines. Individually, the missense variants and insertion were predicted to produce moderate but not deleterious effects on the functionality of the protein (Table S9). However, a global evaluation of these changes with the iTASSER program (Zhang, 2008) predicted the 4 exonic variations to alter the interactions of the residues belonging to the homeodomain region (between 390 and 440) (data not shown). MELO3C014521 contained 3 SNPs and 4 INDELs causing two changes of moderate effect: one missense change (A143P) specific for PS, CAL and VED, and one inframe deletion (D109del) present in all lines except SC. However, no deleterious effect on the functionality of the protein was reported by any of the programs exploited. For MELO3C014522, 23 SNPs and 8 INDELs were also identified, with most of the variants accumulating within intronic regions. Only two variants mapped on exons, resulting in synonymous changes, and 3 variants (including 2 INDELs and 1 SNP) mapped in the UTR region. These changes were predicted to not produce a relevant functional impact on the protein, so the analysis was not extended to the other resequenced lines.

**Figure 5:**
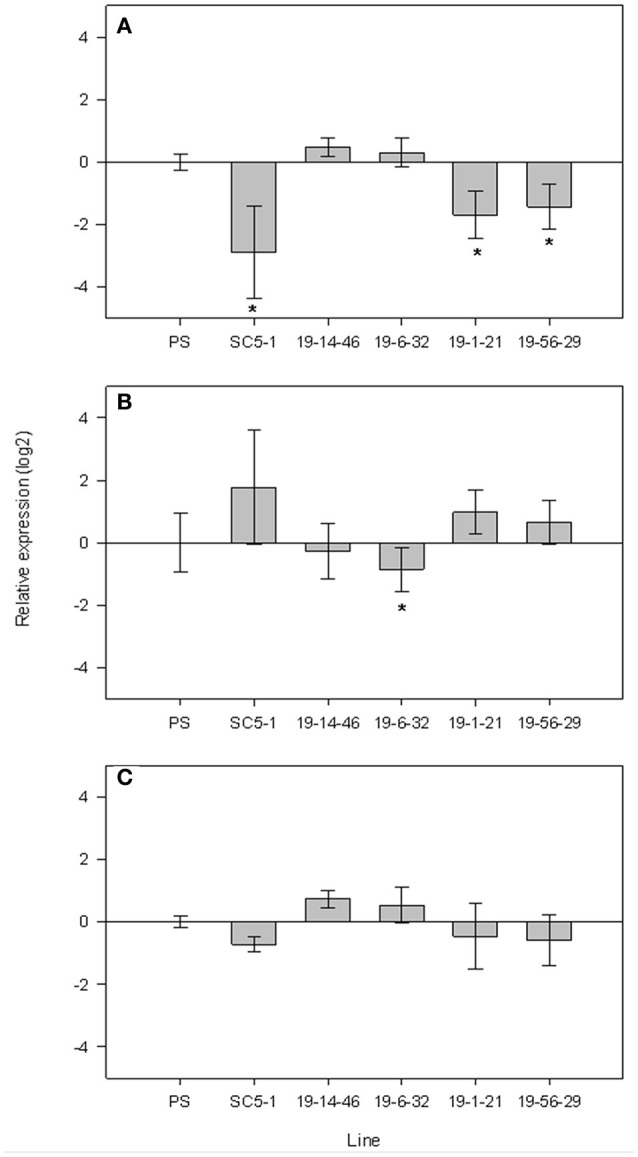
qPCR expression of candidate genes MELO3C014519 **(A)**, MELO3C014521 **(B)** and MELO3C014522 **(C)** located in the *sscqsc5.1* and *sucqsc5.1* intervals in 55 DAP fruits of high and low sugar F_4_ subNILs. Error bars show ±SE of five biological replicates. Lines showing significant differences in relative expression (^*^) with Dunnett test at *p* < 0.5.

When analyzing the expression patterns in mature fruits of the SC subNILs and parental lines harvested at 55 DAP relative to PS, MELO3C014519 showed a nearly 6-fold decrease in expression in SC5-1, and was also downregulated in the low-sugar subNILs 19-1-21 and 19-56-29 by 2.7- and 3.2-fold, respectively (Figure 5A). Compared to SC5-1 and the low sugar lines, gene expression was significantly upregulated in the high sugar lines 19-14-46 and 19-6-32, with a 1.2–1.4-fold increase in expression relative to PS, respectively. MELO3C014521 was more variable and showed the opposite pattern of MELO3C014519, being upregulated relative to PS in the low sugar parent and the low sugar subNILs, and downregulated in the high sugar lines (Figure 5B). Only in 19-6-32 was the decrease in expression significant, however. Expression of MELO3C014522 changed little relative to PS, and did not differ significantly among the lines (Figure 5C).

## Discussion

### *SUCQSC5.1* is a stable QTL on melon chromosome 5 reducing SSC and SUC

Sugar accumulation in melon fruit flesh is a complex, multigenic trait that is highly affected by environmental conditions and agronomic practices (del Amor et al., 1999; Beaulieu et al., 2003; Kano, 2006). The QTL detected in studies of the trait often account for low levels of phenotypic variation, are unstable across years, and show strong genotype-by-environment interactions which further limit efforts to understand its genetic regulation. (Monforte et al., 2004; Eduardo et al., 2007; Paris et al., 2008; Obando-Ulloa et al., 2009; Perpiña et al., 2016). The use of multiple populations to map QTL involved in sugar accumulation, allowed us to overcome some of these limitations and identify consensus QTL collocating to an interval of 400 kb from approximately 1.3–1.7 Mb. on chromosome 5 (Figure 1). Given that in other instances NILs in this study sharing a common SC interval were detected together for the same traits (e.g., SC4-1 and SC4-3; SC7-3 and SC7-4; SC8-1 and SC8-2) (Table 2), it is likely that the SC5-1 and SC5-2 pair represented single stable QTL (*sscqsc5.1* and *sucqsc5.1*) that reduced SSC and SUC by an average of 29 and 62%, respectively.

Importantly, this interval also collocates with QTL described in previous studies for reduced SSC (*SSCQA5.1, SSCQA5.1A, SSCQC5.2*) and increased fructose and glucose content (*FRUQH5.2, GLUQH5.2*) in three different populations derived from PS × SC (F2, DHL, NIL) (Diaz et al., 2011; Table 5). SSC is commonly used to predict sugar content, and has been shown to have a high correlation to sucrose accumulation (Stepansky et al., 1999; Harel-Beja et al., 2010). Thus, we hypothesize that all of the collocating QTL in the PS × SC lines are in fact a single consensus QTL consistently reducing sugar accumulation in melon fruit that we named *SUCQSC5.1*.

**Table 5:** QTL related to sugar accumulation detected in other studies collocating to clusters detected in SC NILs on chromosomes 4, 5, and 7.

**Cultivar or accession, sub-species, and group**		**DP origin**	**Gen**	**References**	**QTL^a^^,^^b^**	**Start interval (Mb)**	**End interval (Mb)**
**Background parent**	**Donor parent (DP)**						
Piel de Sapo (PS) “T111” (ssp. *melo inodorus*)	“Songwhan Charmi” (SC) (ssp. *agrestis conomon)*	Korea	F2/DHL	Monforte et al., 2004	*SSCQA5.1↓*	1.51	2.41
					*SSCQA5.1A↓*		
			NIL	Eduardo et al., 2007	*SSCQC4.4↓*	20.5	30.9
					*SSCQC5.2↓*	0	19.9
					*SSCQC7.2↓*	21.9	25.4
				Obando-Ulloa et al., 2009	*GLUQH4.1*↑	1.2	10.6
					*FRUQH5.2↑*	0	19.9
					*GLUQH5.2↑*	0	19.9
					*FRUQH7.4↑*	22.5	25.4
“Dulce” (spp. *melo reticulatus*)	PI 414723 (spp. *agrestis momordica)*	India	RIL	Harel-Beja et al., 2010	*GLUQN4.1↑*	27.2	30.9
			RIL		*SSCQN5.1↓*	1.7	1.73
			RIL		*SUCQN5.1↓*	1.7	1.73
Piel de Sapo (PS) “T111” (ssp *melo inodorus*)	PI 177362 “Queen Anne's Pocket” (ssp *agrestis dudaim*	Iraq	IL	Castro et al., 2017	*ssc5↓*	0	1.7
Top Mark (ssp *melo cantalupensis*)	USDA-846-1 (ssp *agrestis* and *melo* mix)		RIL	Paris et al., 2008	*SSCQJ7.1↓*	12.2	13.3

a *QTL nomenclature adapted from Diaz et al. (2011)*.

b *↓ or ↑ indicate effect of DP allele on trait value*.

*See main text for references*.

### Colocation with QTL in other populations and considerations for domestication

*C. melo* was first domesticated in India (Sebastian et al., 2010). The species then underwent an extensive process of diversification for a number of fruit characteristics, including sweetness (Monforte et al., 2014), during an East to West geographical differentiation into two subspecies; where Occidental accessions comprise the main part of the ssp *melo*, while Oriental accessions comprise the majority of the ssp *agrestis* (Serres-Giardi and Dogimont, 2012). Melons within botanical groups of the former are generally high in sugar, while those of the latter are generally low in sugar (Pitrat, 2016). It is interesting then that *SUCQSC5.1* colocated with *sscqt5.1* and *sucqt5.1* (Figure 1) and also with QTL described in two other populations (Table 5). In the first instance were QTL reducing SSC and SUC (*SSCQN5.1* and *SUCQN5.1*) identified in RILs derived from the cross of the cultivar “Dulce” (*C. melo* ssp. *melo* group *reticulatus*) × PI 414723 (*C. melo* ssp. *agrestis* group *momordica*) (Harel-Beja et al., 2010). The accession PI 414723 is probably descended from the Indian traditional variety “Kjira” (McCreight et al., 1992). In the second instance was a QTL reducing SSC (*sscqd5.1*) in an advanced backcross generation line derived from PS × PI 177362 (*C. melo* ssp. *agrestis* group *dudaim*) or Queen Anne's Pocket Melon (Castro et al., 2017) which is native to the Middle East.

The collocation of QTL of consistent effect in the same genomic region as *SUCQSC5.1*, suggests that they are allelic. That the ssp *agrestis* allele decreased sugar content in crosses involving sweet melon cultivars of Occidental origin and wild, or non-sweet melons of Oriental origin (Tables 2, 3, 5) also raises the possibility that the native allele did not induce higher sugar accumulation in the fruit flesh. In fact, in the analysis of resequenced lines of both subspecies described in Sanseverino et al. (2015) representing high or low sugar types (Stepansky et al., 1999) we confirmed that the missense variants and in-frame insertion predicted to affect protein function of MELO3C014519, a candidate gene for *SUCQSC5.1* (discussed in more detail below), are restricted to the PS and VED alleles (ssp. *melo*, high-sugar, Occidental cultivars) and are absent in SC, CAL, TRI, and CV alleles (Figure 4) (Table S9). (ssp. *agrestis*, low-sugar, Oriental landraces and wild accessions). After domestication, likely no mutation affecting protein function occurred in the linage that ended in the melons from the *agrestis* ssp. which maintained lower sugar accumulation, whereas mutations did occur in some of the Occidental cultivar lineages during the process of diversification. This helped lead to sweeter melons found in ssp. *melo*, particularly in the *inodorus* and *cantalupensis* groups. Further research is needed to confirm this hypothesis, such as the sequencing of the candidate genes contained in *SUCQSC5.1* in the melon germplasm collection (Leida et al., 2015) to determine if the variants are restricted to ssp. *melo*.

### Additional QTL detected in the PS × SC NILS and TRI-derived lines

Excluding the cluster on chromosome 5, we identified 48 additional QTL (14 for SSC, 27 for soluble sugars, and 7 for organic acids) for 6 traits in the set of 47 SC NILs (Table 2) and 11 QTL (6 for SSC, 2 for soluble sugars, and 3 for organic acids) for 5 traits in the TRI mapping populations (Table 3). The number of QTL identified for SSC and soluble sugars in the SC NILs are similar to what was reported previously using fewer lines (Eduardo et al., 2007; Obando-Ulloa et al., 2009). Thus, the inclusion of more NILs in this work did not result in the detection of significantly more QTL. However, it did provide redundancy which helped to more accurately define QTL intervals when SC introgressions overlapped (e.g., chromosomes 4, 5, and 7), and assuming a QTL collocated to the shared interval, more replicates to better estimate its effects.

We encountered limitations described in other studies in mapping QTL for a highly variable trait like sugar accumulation in identifying a large proportion whose effects were poorly consistent among independent experiments in the SC NILs and TRI derived-lines (Tables 2, 3). Nonetheless, the less stable QTL (two environments) for sugars detected on chromosomes 4 and 7 also collocated in some cases with QTL described in previous studies (Table 5) so could make interesting targets for future investigations. For example, the cluster of six QTL on chromosome 4 colocates with a QTL previously detected in NILs for reduced GLC content (Obando-Ulloa et al., 2009). An annotated gene for hexokinase like enzyme 1 (*CmHK-LIKE1*) collocated within the common QTL interval (Table S1). Also, the nine QTL detected on chromosome 7 collocate with two affecting SSC (*SSCQJ7.1, SSCQC7.2*), and a third affecting FRU (*FRUQH7.4*). Furthermore, sucrose metabolism related genes UDP-glucose epimerase 2 (*CmUGE2*) and fructokinase-like 3 (*CmFK-LIKE3*) collocated to the interval shared between SC7-3 and SC7-4 (Table S1).

As mapping studies in melon have been conducted between elite, high-sugar cultivars, and wild accessions or landraces, favorable QTL alleles increasing SSC or SUC content beyond the already high levels of the former have not been described (Eduardo et al., 2007; Paris et al., 2008; Obando-Ulloa et al., 2009; Park et al., 2009; Harel-Beja et al., 2010; Castro et al., 2017). Our results were similar in that we identified just one QTL increasing SSC in one location (*sscqsc3.5_VAL11*) in NIL SC3-5. This line was previously identified as having significantly increased SSC in two locations (Eduardo et al., 2007), which could make it a potential target for fine-mapping, although SUC content was also not significantly increased (Obando-Ulloa et al., 2009). Recently, QTL detected in lines containing introgressions from the Japanese cultivar “Makuwa” (*C. melo* ssp. *agrestis*) have been reported, representing the first instances in which exotic alleles increasing sugar content have been identified (Perpiña et al., 2016).

For the organic acids, both NILs SC8-1 and SC8-2 showed significantly reduced citrate in CDM and ZA11 in the TRI F_2_ population. The PH gene, which determines fruit acidity in melon, located between 1.07 and 1.08 Mb on chromosome 8, also falls outside of this interval (Cohen et al., 2014). However, as the magnitude of the effect of the QTL was very large in both SC and TRI, it could also be a target for further investigation.

### Fine mapping of *SUCQSC5.1* in subNILs

*SUCQSC5.1* was fine mapped to a 56.1 kbp interval from 1,698 to 1,754 kbp on chromosome 5 where it significantly reduced SSC and SUC by 18 and 34%, respectively in the SC subNILs (Figures 3B,C). The effect of the QTL was less than in the NILs (29 and 62% for SSC and SUC, respectively). However, many environmental factors can influence sugar accumulation in melon, including temperature, nutrition, and the season of production (del Amor et al., 1999; Beaulieu et al., 2003). A large effect of location (LOC) on total variance for both SSC and SUC in the subNILs (Table S5) highlighted this, so the differences in the magnitude of the QTL effects between the generations may have been due to year-to-year environmental variation. Another explanation is possible epistatic or additive interactions between the QTL and either the genes in the 4.4 Mb introgression on chromosome 9 that SC5-1 and SC5-2 shared (Table S1), or a second, tightly linked QTL in the original interval as suggested in the F_2_ generation (Figure 2C). In the former case, it is impossible to assess as there were not any NILs containing only SC introgressions on chromosome 5 or chromosome 9 absent the other. In the latter, as none of F_4_ subNILs contained SC alleles in both of the putative QTL regions, it would be necessary to include more lines in a future genetic analysis to examine possible interactions between the loci.

We failed to detect significant differences in hexose content in the subNILs despite the collocation of QTL for increased hexose and reduced SUC and SSC in the NILs in this and previous studies (Tables 2, 5). This supported the idea that the *SUCQSC5.1* allele affects sucrose accumulation independently of hexose accumulation as described in other melon genotypes (Stepansky et al., 1999).

### Candidate genes for *SUCQSC5.1*

The *SUCQSC5.1* interval contained four candidate genes (MELO3C014519-MELO3C014522) (Table 1), with MELO3C014520 discarded after determining that it was not expressed in fruit tissue. Regarding the remaining three genes, there are at least 10 BEL1-like (MELO3C014519), one FAF-like (MELO3C014521) and 13 BSL2-like (MELO3C014522) homologs in melon. In *Arabidopsis*, BEL1 is necessary for correct ovule development and patterning through the establishment of auxin and cytokinin signaling pathways (Reiser et al., 1995; Bencivenga et al., 2012). In potato (*Solanum tuberosum*), a BEL1-like protein (*StBEL5*) predicted to be a close homolog of MELO3C014519 (http://phylomedb.org) works in a transcriptional complex to activate tuber growth via the regulation of gibberellin levels in stolon tips (Chen et al., 2003, 2004). A BEL1-like homeodomain gene *Malus domestica* homeobox gene 1 (*MDH1*) is also involved in the early stages of fruit growth and in the determination of fruit shape in apple *(Malus domestica* Borkh), (Dong et al., 2000). The FAF class of genes is plant specific. In *Arabidopsis* there are four members plus a more distantly related FAF-like which are postulated to interact with the homeodomain transcription factors WUSCHEL (WUS) and CLAVATA (CLV) (Wahl et al., 2010) to determine the size of the shoot apical meristem. They are most strongly expressed in the vasculature and are responsive to cytokinin (Brenner et al., 2012). BSL2 belongs to a family of serine/threonine-protein phosphatases containing N-terminal kelch-repeats and function in brassinosteroid signaling in *Arabidopsis* (Mora-Garcia et al., 2004; Farkas et al., 2007). None of these genes are directly involved in sucrose metabolism in melon (Dai et al., 2011), and have not been implicated in sugar accumulation in other species. Perhaps this is not surprising, as previous studies in both melon and tomato have found a general absence of colocalization between QTL for sugar content and genes encoding enzymes involved in primary sugar metabolism (Bermudez et al., 2008; Harel-Beja et al., 2010). This implies a role for other genes in sugar accumulation, as have been described in tomato (Ariizumi et al., 2011; Sagar et al., 2013; Sauvage et al., 2014).

### Possible role for MELO3C014519

As incoming sugars to the fruit sink are partitioned between growth and storage, factors affecting growth of the fruit sink have the potential to alter the proportion of incoming sugars directed to storage (sugar accumulation), thereby producing an indirect but significant effect on sucrose accumulation. MELO3C014519 showed a differential expression pattern in 55 DAP fruits consistent with the high or low sugar phenotype of subNILs (Figure 5) and contained three missense changes and 1 in-frame deletion present in alleles of the low sugar lines (SC and TRI) that combined were predicted to alter protein confirmation and interactions in the homeodomain region (Figure 4A). Since highly conserved homeodomain regions are essential for correct DNA binding process/function, these changes could lead to an altered recognition of the downstream genes activated/repressed by the BEL-1 transcription factor and a feedback effect on expression due to altered activity of downstream genes. Given that BEL1-like genes are involved in the formation and growth of the photoassimilate sink in other species, and the correlation between expression differences, sequence variation and phenotype, it is plausible that MELO3C014519 is also involved in sugar accumulation in melon by affecting growth processes.

Some evidence for this comes from a transcriptomic analysis comparing NIL SC5-1 to PS fruit at four developmental stages (25, 35, 45, and 55 DAP) (Argyris et al., unpublished). Differentially expressed genes enriched in the functional categories related to growth of cell wall and cellulose synthesis were significantly upregulated in SC5-1 at 35 and 45 DAP, while those for cell wall degradation were significantly downregulated at 45 DAP (Figure S1). This is much later than when melon fruits begin to accumulate significant amounts of sucrose (25–30 DAP) (Saladie et al., 2015) that typically marks the end of the growth phase (Dai et al., 2011). The ssp. *agrestis* allele of MELO3C014519 may then function in prolonging this phase, redirecting the flux of sugars toward growth processes (i.e., cell wall synthesis) instead of storage. Given its involvement in ovule patterning in *Arabidopsis* and the early growth of both potato and apple, it is surprising that expression of MELO3C014519 is apparent in later stages in fruit development. However, it was expressed at all stages of development, and significantly downregulated at both 35 and 55 DAP in the transcriptomic study (Figure S2). Furthermore, BEL1-like transcripts present at the turning stage of ripening fruits in tomato have also been identified (Bartley and Ishida, 2003), lending support to a continuing role for the gene during maturation.

### Possible involvement of other candidate genes

Compared to MELO3C014519, expression differences for MELO3C014521 and MELO3C14522 in the high and low sugar lines were smaller and not significant (Figures 4B,C) and sequence variations potentially affecting protein function less clear-cut (Figures 4B,C, Table S9). There is less known about the FAF genes in plants, thus a hypothesized role for MELO3C014521 in sugar accumulation in melon is also less clear. Wahl et al. (2010) did speculate that FAF genes could act in translating sugar signals to meristem maintenance, thus providing a possible link to sugar metabolism. Brassinosteroids also play a diverse role in plant growth and development (Yang et al., 2011), thus could hypothetically act to affect some aspect of sugar accumulation through MELO3C014522 in melon fruit as well. As differences solely in gene expression or sequence variation do not exclude other possible regulatory mechanisms like post-transcriptional modification, none of the three genes can be ruled out in affecting SSC or sucrose content. Additional studies such as their spatial and temporal expression in floral organs and developing fruit, histological studies, and analyses of their function by random (TILLING) or directed (CRISPR-Cas9) mutagenesis, are needed to confirm the identity of *SUCQSC5.1*.

### Impact on plant breeding

Sugar accumulation in melon fruit flesh has been reported to have low heritability and strong GxE interactions (Monforte et al., 2004; Eduardo et al., 2007; Paris et al., 2008; Obando-Ulloa et al., 2009; Perpiña et al., 2016) that complicates breeding initiatives to improve the trait. Nevertheless, we identified the stable QTL *SUCQSC5.1* and fine mapped it to an interval containing genes possibly representing families previously unknown to affect sugar accumulation. Through fine-mapping with the subNILs we could accurately estimate its phenotypic effect and provide a hypothesis for its function. These results provide new insights into the mechanisms of sugar accumulation in melon. In spite of the potential biological/physiological relevance of these findings, the ssp *agrestis* alleles of *SUCQSC5.1* derived from SC and TRI, as well as its putative alleles *SSCQN5.1* (Harel-Beja et al., 2010) and *sscd5.1* (Castro et al., 2017) reduce sugar content, meaning that traditional farmers probably fixed the allele increasing sugar accumulation in the Occidental sweet melons. Some evidence comes from the presence of selection signatures at loci close to *SUCQSC5.1* that were identified between group *chate* (low sugar) and *inodorus* melons (Pavan et al., 2017). Therefore, from the applied breeding point of view, the wild allele has no value. However, the molecular markers linked to the QTL developed in this work can be used in breeding programs with wild accessions to select against those alleles reducing SSC. Furthermore, the identification of candidate genes and corresponding polymorphisms could help to create new alleles through mutagenesis that would aid crop improvement.

## Author contributions

JA and AD cultivated, selected and phenotyped the SC NILs, subNILs and TRI lines; performed genotyping and statistical analysis, and participated in the design of the study. JA performed qPCR analyses and drafted the manuscript. VR performed the resequencing and variant analyses. MF and TJ cultivated and analyzed the SC NILs in CAM. YG performed the analyses of soluble sugars and organic acids, and interpretation of data. BP directed cultivation of the SC NILs, subNILs, and TRI lines in VAL, and provided analyses and access to additional data. AMM cultivated the NILs in CDM, and performed crosses to generate the subNILs. AJM and JG conceived, designed and coordinated the study, and helped to draft the manuscript.

### Conflict of interest statement

The authors declare that the research was conducted in the absence of any commercial or financial relationships that could be construed as a potential conflict of interest.

## Supplementary material

Figure S1 PageMan display of DEGs belonging to significantly changed pathways throughout melon fruit development. BINs colored in red are significantly up-regulated in SC5-1 relative to other BINs in the RNAseq experiment, whereas BINs colored in blue are down-regulated.

Figure S2 qPCR expression of MELO3C014519 in SC5-1 (light bar) relative to PS (dark bar) at 55 days after pollination (DAP) over four stages of development. Error bars show ±SE of three biological replicates.
