# Supplementary figures and images for: QTL Analyses in Multiple Populations Employed for the Fine Mapping and Identification of Candidate Genes at a Locus Affecting Sugar Accumulation in Melon (Cucumis melo L.)
Source: Front Plant Sci. 2017 Sep 26;8:1679. doi: 10.3389/fpls.2017.01679 (PMC5623194; doi:10.3389/fpls.2017.01679)

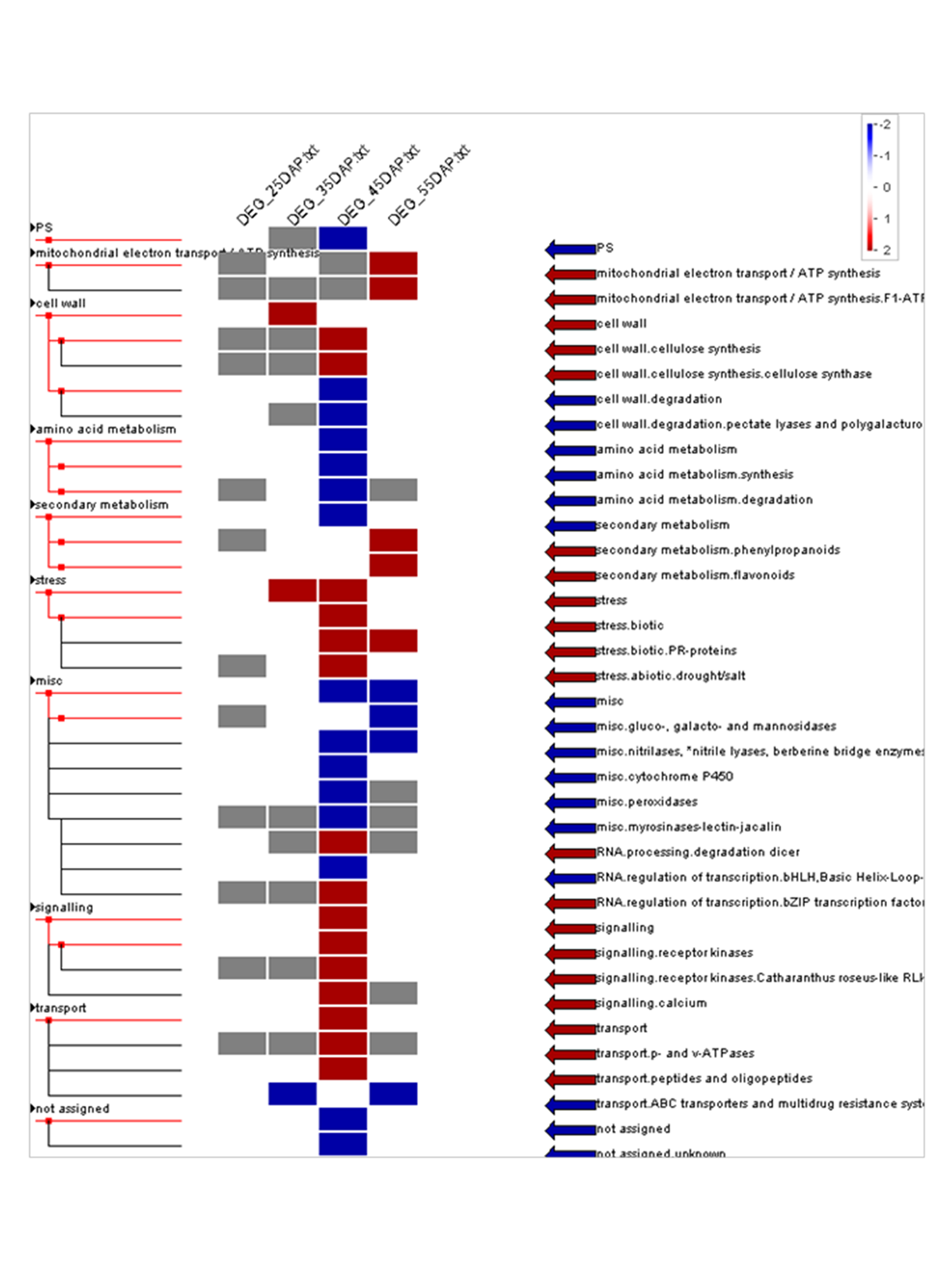

Supplement: Figure S1 — PageMan display of DEGs belonging to significantly changed pathways throughout melon fruit development. BINs colored in red are significantly up-regulated in SC5-1 relative to other BINs in the RNAseq experiment, whereas BINs colored in blue are down-regulated. [file Image1.TIF]

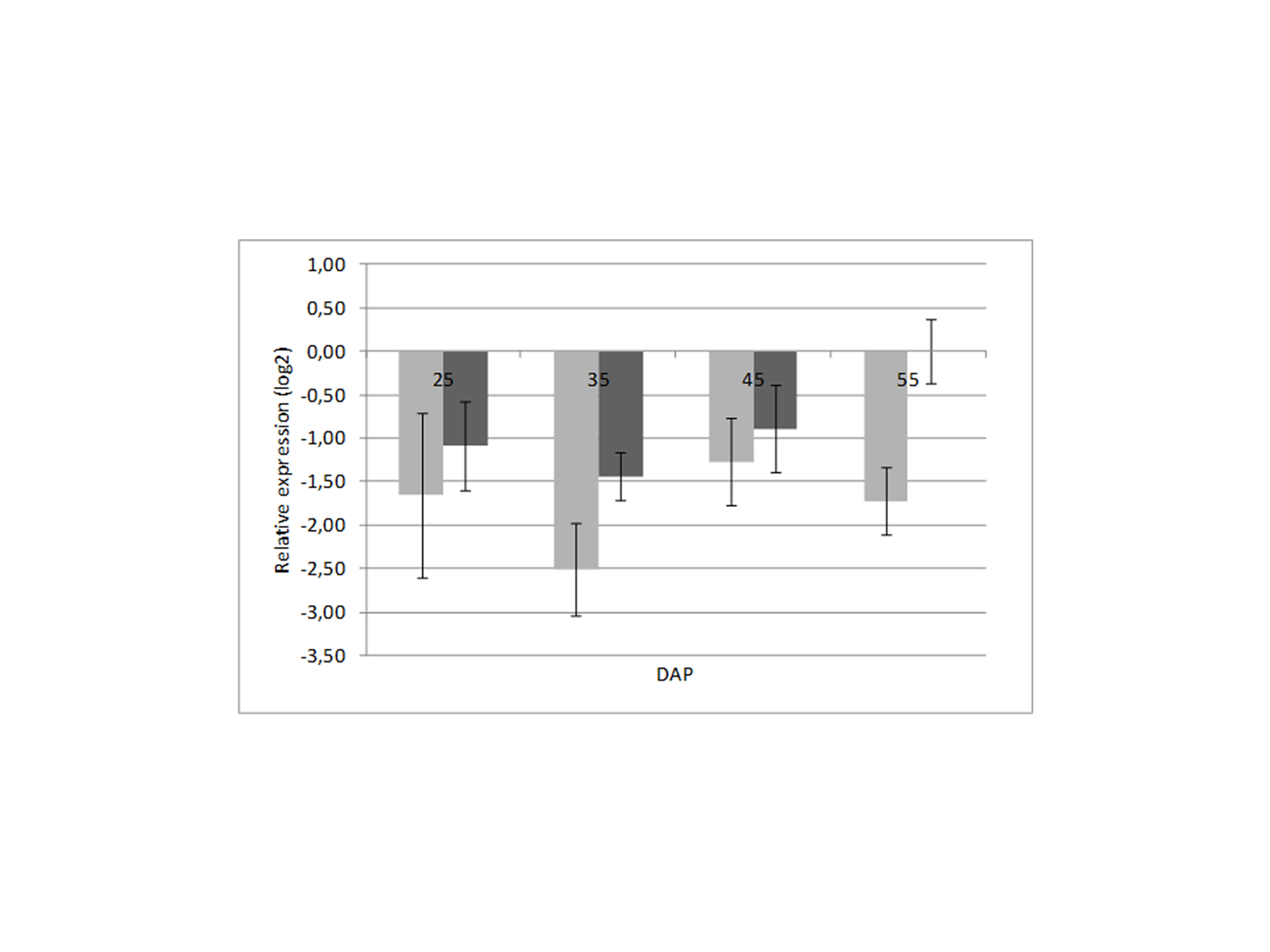

Supplement: Figure S2 — qPCR expression of MELO3C014519 in SC5-1 (light bar) relative to PS (dark bar) at 55 days after pollination (DAP) over four stages of development. Error bars show ±SE of three biological replicates. [file Image2.TIF]
